# Causal Representation Learning from Multi-modal Biomedical Observations

**Published:** 2025-03-16

**Authors:** Yuewen Sun, Lingjing Kong, Guangyi Chen, Loka Li, Gongxu Luo, Zijian Li, Yixuan Zhang, Yujia Zheng, Mengyue Yang, Petar Stojanov, Eran Segal, Eric P. Xing, Kun Zhang

**Affiliations:** 1 Mohamed bin Zayed University of Artificial Intelligence; 2 Carnegie Mellon University; 3 University of Bristol; 4 Broad Institute of MIT and Harvard

## Abstract

Prevalent in biomedical applications (e.g., human phenotype research), multi-modal datasets can provide valuable insights into the underlying physiological mechanisms. However, current machine learning (ML) models designed to analyze these datasets often lack interpretability and identifiability guarantees, which are essential for biomedical research. Recent advances in causal representation learning have shown promise in identifying interpretable latent causal variables with formal theoretical guarantees. Unfortunately, most current work on multi-modal distributions either relies on restrictive parametric assumptions or yields only coarse identification results, limiting their applicability to biomedical research that favors a detailed understanding of the mechanisms.

In this work, we aim to develop flexible identification conditions for multimodal data and principled methods to facilitate the understanding of biomedical datasets. Theoretically, we consider a nonparametric latent distribution (c.f., parametric assumptions in previous work) that allows for causal relationships across potentially different modalities. We establish identifiability guarantees for each latent component, extending the subspace identification results from previous work. Our key theoretical contribution is the structural sparsity of causal connections between modalities, which, as we will discuss, is natural for a large collection of biomedical systems. Empirically, we present a practical framework to instantiate our theoretical insights. We demonstrate the effectiveness of our approach through extensive experiments on both numerical and synthetic datasets. Results on a real-world human phenotype dataset are consistent with established biomedical research, validating our theoretical and methodological framework.

## 1 Introduction

Multimodal datasets provide rich and comprehensive insights into complex biomedical systems, offering the potential to provide a deeper understanding of physiological mechanisms. For example, the human phenotype dataset (Levine et al., 2024) contains measurements from multiple modalities, including anthropometric data, sleep monitoring, and genetic information. Proper analysis of such data can potentially uncover the underlying mechanisms that drive phenotypic diversity and disease susceptibility, leading to the discovery of novel molecular markers and the development of predictive models for disease. Recent advances in large-scale models have made it possible to exploit large biomedical datasets for various tasks such as protein structure prediction (Jumper et al., 2021; Lin et al., 2023), gene-disease association identification (Diaz Gonzalez et al., 2023; Zagirova et al., 2023), and novel drug candidate discovery (Pal et al., 2023; Zheng et al., 2024b).

Despite the impressive performance of these models, their trustworthiness remains a contentious issue (Zheng et al., 2023). A major concern lies in their lack of interpretability, which poses significant challenges in biomedical research and hinders the safe and ethical application of these models. For example, in clinical decision-making (Hager et al., 2024), if the model recommends a specific treatment plan for a patient, it is important for clinicians to understand the rationale behind the recommendation. Without such transparency, it is difficult to trust the model’s output or integrate these systems into critical decision-making workflows. Although several explainable models have been developed for multimodal datasets (Tang et al., 2023), this area remains largely underexplored.

Fortunately, recent advances in causal representation learning (CRL) (Schölkopf et al., 2021) have shown promise in identifying latent causal structures from raw observations, making it well-suited for biomedical applications. For example, a plethora of CRL studies (Hyvarinen et al., 2019; Khemakhem et al., 2020a; Zhang et al., 2024b; Buchholz et al., 2024; von Kügelgen et al., 2023; Zhang et al., 2024a; Li et al., 2024c; Ahuja et al., 2023) effectively utilize temporal information or domain indices to identify latent causal models and apply them in fMRI data. Recently, a growing body of CRL research has investigated multimodal distributions (Yao et al., 2023; Morioka & Hyvarinen, 2023; 2024; Daunhawer et al., 2023; Sturma et al., 2023; Gresele et al., 2020). These works leverage shared information across modalities to establish identifiability guarantees for latent variables (Yao et al., 2023; Morioka & Hyvarinen, 2024; Daunhawer et al., 2023). Despite these advancements, some aspects of these works are still limited. For instance, Von Kügelgen et al. (2021); Daunhawer et al. (2023); Yao et al. (2023) only focus on identifying latent subspaces that are directly shared by multiple modalities. In practice, however, many informative latent variables may influence multiple modalities indirectly through intermediate latent mechanisms. Moreover, such subspace identifiability loses track of the intricate causal influences among individual components, leading to a limited view of the underlying latent mechanism. Morioka & Hyvarinen (2024); Gresele et al. (2020); Morioka & Hyvarinen (2023) rely on specific assumptions about latent variable distributions (e.g., independence or exponential family). These constraints significantly limit their applicability for biomedical datasets that involve complex interactions among latent factors.

**Figure 1:**
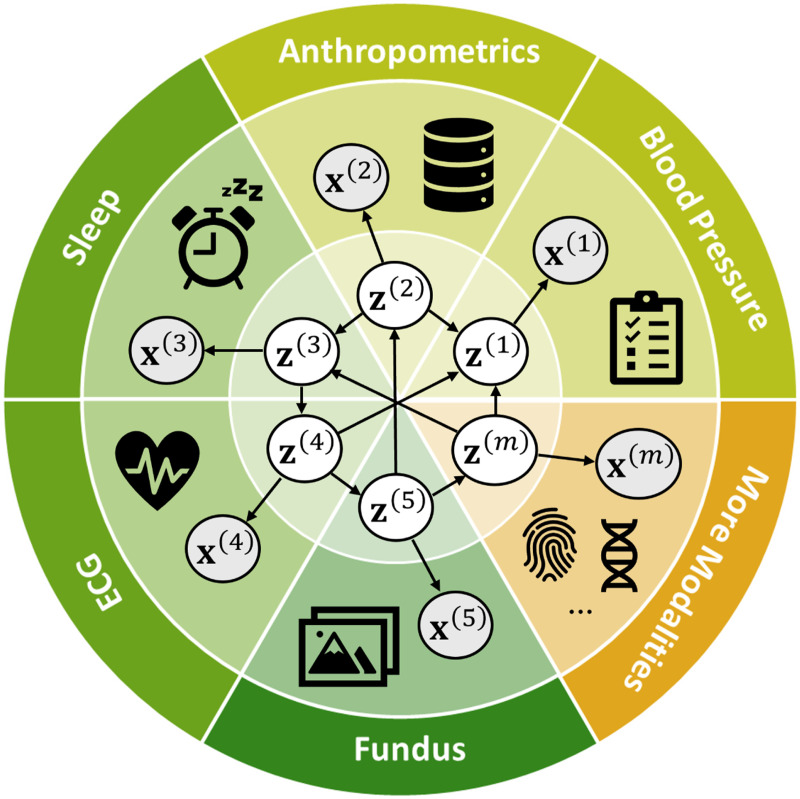
Multimodal data with causal latent variables.

In this work, we aim to develop identification theory with *multimodal biomedical* datasets in mind, and design *principled and interpretable* models to facilitate analyzing such datasets. We assume that observations ${\text{x}}^{\left(m\right)}$ in each modality m are generated by a specific set of latent components ${\left\{z_{i}^{\left(m\right)}\right\}}_{i}$, and allow for flexible causal relationships among latent components from potentially different modalities, such that $z_{i}^{\left(m\right)}\to z_{j}^{\left(n\right)}$ for m≠n, i≠j, as shown in Figure 1. **Theoretically**, we provide identifiability guarantees for each latent component $z_{i}^{\left(m\right)}$, thus generalizing the subspace identification results in Yao et al. (2023); Daunhawer et al. (2023) while avoiding independence or parametric restrictions on the latent distribution $p\left({\left\{{\text{z}}^{\left(m\right)}\right\}}_{m}\right)$ as in Morioka & Hyvarinen (2023; 2024); Gresele et al. (2020). In particular, we first show that any latent subspace ${\text{z}}^{\left(m\right)}$ can be identified as long as ${\text{z}}^{\left(m\right)}$ exerts sufficient influences on other modalities, which is weaker than assuming that ${\text{z}}^{\left(m\right)}$ is directly shared across multiple modalities as in Daunhawer et al. (2023); Yao et al. (2023). Based on this subspace identification, we leverage the sparsity of the causal connections between modalities to further identify each latent component ${\left\{z_{i}^{\left(m\right)}\right\}}_{i}$. This notion of causal sparsity has been explored in recent work (Lachapelle et al., 2023; Xu et al., 2024; Zheng et al., 2022) in other causal identification settings and has been shown realistic in many biomedical systems (Busiello et al., 2017; Milo et al., 2002; Babu et al., 2004; West et al., 2002;Banavar et al., 1999), as we will discuss further in Section 4.

**Empirically**, we develop a theoretically grounded estimation framework to recover the latent components in each modality. Our model implements our theoretical conditions (in particular, conditional independence and sparsity constraints) on top of normalizing flow (Huang et al., 2018; Kobyzev et al., 2020) within the encoder-decoder framework. Extensive experiments on both numerical and synthetic datasets demonstrate its effectiveness. Most notably, our framework enables the identification of latent causal variables that capture complex biomedical interactions and facilitates the analysis of potential causal mechanisms across modalities, which are important for clinical decision-making. The evaluation results on a real-world human phenotype dataset provide novel insights into the relationships between modalities, and the discovered causal relationships align with the findings from biomedical research, highlighting our contributions to the biomedical domain.

## 2 Related Work

### ML models for biomedical research.

For biomedical applications, ML models are developed to extract informative representations to facilitate downstream tasks, including DNA sequence modeling (Zhou et al., 2024; Nguyen et al., 2023;Dalla-Torre et al., 2023), protein structure prediction (Jumper et al., 2021; Lin et al., 2023), and disease detection (Zhou et al., 2023; Jang et al., 2024). The success of large language models (LLMs) has significantly advanced sequence modeling for DNA, RNA, and proteins (Celaj et al., 2023; Shulgina et al., 2024; Nguyen et al., 2024; Li et al., 2023; Chen et al., 2023; Lin et al., 2023), yet these methods primarily operate on a single modality, limiting their applicability to the multimodal datasets, which are commonly encountered in biomedical research. Although several studies have explored integrating multimodal biomedical data (Garau-Luis et al., 2024; Pei et al., 2024; Taylor et al., 2022), these approaches often lack theoretical guarantees, raising concerns about the reliability of their results. In this paper, we leverage causal principles to develop theoretically sound ML models for multimodal biomedical data, aiming to provide reliable and interpretable insights into complex biomedical systems.

### Multimodal representation learning.

Multimodal representation learning (Zhang et al., 2020; Manzoor et al., 2023) refers to the process of learning representations from multiple data modalities (e.g., text, image, audio) for specific tasks. Recent advances have leveraged contrastive learning techniques to improve the alignment of latent spaces across different modalities (Daunhawer et al., 2023; Wang et al., 2022; Radford et al., 2021; Khosla et al., 2020). Methods like CLIP (Radford et al., 2021) and Contrastive Predictive Coding (Oord et al., 2018) have demonstrated the ability to recover shared latent factors across modalities by (implicitly) maximizing mutual information between representations. However, challenges remain in achieving finding modality-specific representations, which requires novel approaches that preserve the unique characteristics of each modality.

### Identifiable CRL.

CRL aims to identify high-level causal variables from low-level observations, integrating principles from both machine learning and causality (Schülkopf et al., 2021), and can be viewed as an extension of causal discovery (Spirtes et al., 2001; Li et al., 2024a; Luo et al., 2025; Li et al., 2024b; Ziu et al., 2024). CRL methods with identifiability guarantees can be classified based on the assumptions they impose, including sparsity constraints (Xu et al., 2024; Zheng et al., 2022; Zheng & Zhang, 2023; Lachapelle et al., 2024), interventional/multi-distribution settings (Hyvarinen et al., 2019; Khemakhem et al., 2020a; Zhang et al., 2024b; Kong et al., 2023; Buchholz et al., 2024; von Kügelgen et al. , 2023; Zhang et al., 2024a; Li et al., 2024c; Varici et al., 2023; Ahuja et al., 2023; Jiang & Aragam, 2023), and of particular relevance to our work, multimodality (Yao et al., 2023; Morioka & Hyvarinen, 2023; 2024; Daunhawer et al., 2023; Sturma et al., 2023; Gresele et al., 2020). To provide a clearer comparison, Table 1 summarizes representative works in the multimodality category and highlights their differences from our work.

### Empirical CRL for multimodal applications.

In contrast to the previously discussed works that emphasize identifiability, another line of multimodal CRL research prioritizes practical applications in various domains, without addressing theoretical identifiability. Mao et al. (2022) assume independent latent variables and introduce a two-module amortized variational algorithm to learn representations from medical images and biomedical data. Zheng et al. (2024a) develop a contrastive learning-based approach to extract modality-specific and modality-invariant representations from time-series tabular and textual data for root cause analysis. Rawls et al. (2021) leverage behavioral and psychiatric phenotyping alongside high-resolution neuroimaging data from the Human Connectome Project (Van Essen et al., 2013), and perform greedy fast causal inference (Ogarrio et al., 2016) to investigate causal relations in alcohol use disorder. In contrast, our work establishes formal identification theory and integrates the theoretical insights into our estimation model.

## 3 Latent Multimodal Causal Models

Real-world biomedical datasets often integrate multiple modalities, each characterizing a unique yet interrelated aspect of the subject. For instance, the human phenotype dataset (Shilo et al., 2021) consists of tabular data, time series, images, and text, capturing diverse biomedical measurements such as anthropometrics, sleep monitoring, and genetic information. Understanding the latent factors behind each modality and their interplay can provide valuable insights into underlying biomedical mechanisms, ultimately facilitating the advancement of biomedical technologies. With this goal in mind, we formalize the multimodal data-generating processes as follows.

### Data-generating processes.

**Figure 2:**
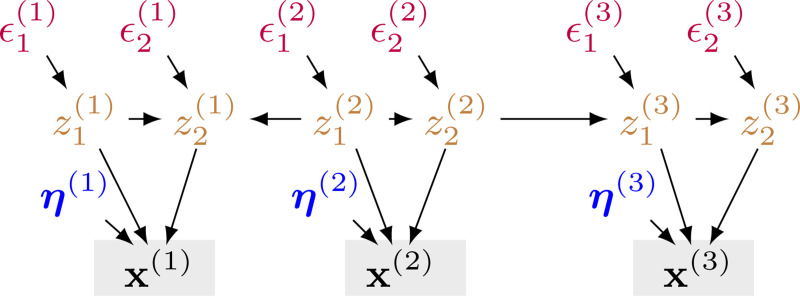
An illustrative example of the hypothesis space underlying the biomedical system.

Let $\text{x}:=\left[{\text{x}}^{\left(1\right)},\ldots ,{\text{x}}^{\left(M\right)}\right]$ be a set of observations/measurements from M modalities, where ${\text{x}}^{\left(m\right)}\in {\mathbb{R}}^{d\left({\text{x}}^{\left(m\right)}\right)}$ represents the observation from modality m with dimensionality $d\left({\text{x}}^{\left(m\right)}\right)$. Let $\text{z}=\left[{\text{z}}^{\left(1\right)},\ldots ,{\text{z}}^{\left(M\right)}\right]$ be the set of causally related latent variables underlying M modalities. Specifically, the data generation process (Figure 2) can be formulated as

(1) $$z_{i}^{\left(m\right)}:=g_{z_{i}^{\left(m\right)}}\left(\text{Pa}\left(z_{i}^{\left(m\right)}\right),\epsilon_{i}^{\left(m\right)}\right),\;(\text{latent}\;\text{causal}\;\text{relations})$$

(2) $${\text{x}}^{\left(m\right)}:=g_{{\text{x}}^{\left(m\right)}}\left({\text{z}}^{\left(m\right)},\eta^{\left(m\right)}\right),\;\;(\text{generating}\;\text{functions})$$

where Pa(⋅) denotes the parents of a variable. Since we allow for causal relations to exist within and across modalities, Pa(⋅) potentially includes latent variables across multiple modalities. The differentiable function $g_{\text{z}}$ encodes the latent causal graph connecting the latent components, and its Jacobian matrix $J_{g_{\text{z}}}$ can be permuted into a strictly triangular matrix. We denote $\epsilon_{i}^{\left(m\right)}$ as the exogenous variable for $z_{i}^{\left(m\right)}$, where all exogenous variables are mutually independent. $\eta^{\left(m\right)}$ represents domain-specific information independent of other components.

### Example.

In healthcare, different modalities capture complementary physiological aspects. A chest X-ray ${\text{x}}^{\left(m\right)}$ may reflect latent factors such as lung density, cardiac silhouette, and ribcage structure, represented by ${\text{z}}^{\left(m\right)}$. These latent factors can causally influence those in other modalities, ${\text{z}}^{\left(n\right)}$, such as pulmonary function parameters (e.g., forced vital capacity) and cardiovascular biomarkers (e.g., left ventricular mass). These, in turn, may affect electrical activity recorded in an ECG, represented by ${\text{x}}^{\left(n\right)}$, by modulating heart rate variability and conduction patterns.

### Goal.

As outlined previously, we aim to learn the latent variables underlying each modality and their causal relations. Formally, consider two specifications of the data-generating process in Eq. (1) and Eq. (2): $\theta :={\left\{g_{{\text{x}}^{\left(m\right)}},g_{{\text{z}}^{\left(m\right)}},p\left(\epsilon^{\left(m\right)}\right)\right\}}_{m=1}^{M}$ and $\hat{\theta }:={\left\{{\hat{g}}_{{\text{x}}^{\left(m\right)}},{\hat{g}}_{{\text{z}}^{\left(m\right)}},\hat{p}\left(\epsilon^{\left(m\right)}\right)\right\}}_{m=1}^{M}$, both of which fit the marginal distribution p(x). Our objective is to show that, *given the same value of* x, each estimated latent component ${\hat{z}}_{i}^{\left(m\right)}$ is equivalent to its true counterpart $z_{i}^{\left(m\right)}$ up to an invertible transformation $h_{i}^{\left(m\right)}$, i.e., ${\hat{z}}_{i}^{\left(m\right)}=h_{i}^{\left(m\right)}\left(z_{i}^{\left(m\right)}\right)$. ^1^This *component-wise identifiability* ensures that latent components (e.g., gene types, nutrient levels) are disentangled from the observed measurements x while preserving their original information. Once component-wise identifiability is achieved, one can readily apply standard causal discovery algorithms (e.g., PC (Spirtes et al., 2001)) to the identified components ${\hat{z}}_{i}^{\left(m\right)}$ to infer the graphical structures. The choice of structure learning algorithms can be tailored to the assumed graph class (e.g., potentially non-DAGs), and this step is orthogonal to our contribution. These structures characterize the interactions between all latent components across modalities, which is particularly desirable for biomedical applications.

## 4 Identification Theory

As motivated in Section 3, we address the component-wise identifiability of latent components $z_{i}^{\left(m\right)}$.

### Remarks on the problem.

Identification for multimodal distributions often leverages the structure among the available modalities. However, component-wise identification, especially in the general nonparametric setting, is challenging. Daunhawer et al. (2023); Von Kugelgen et al.¨ (2021); Yao et al. (2023) require certain information redundancy: the information of the latent variables should be fully shared and preserved by the observations of at least two modalities – that is, we can express ${\text{z}}^{\left(m\right)}$ as functions of ${\text{x}}^{\left(m_{1}\right)}$ and ${\text{x}}^{\left(m_{2}\right)}$ individually. Moreover, the identification can only be achieved up to *subspaces* (i.e., groups of latent components) determined by the sharing pattern. Often, however, the latent components may not be fully shared by multiple modalities. For example, in health monitoring, while sleep monitoring data (e.g., sleep stages or duration) may not fully encode genetic predispositions, genetic factors may still influence sleep disorders, such as insomnia and circadian rhythm disruptions. In this case, the subspace identification may fall short of providing detailed interpretations of biomedical systems and the mechanisms encoded in the graphical structures over individual causal components.

For work that achieves component-wise identifiability, Morioka & Hyvarinen (2023; 2024) assume that the latent distribution $p\left({\left\{{\text{z}}^{\left(m\right)}\right\}}_{m=1}^{M}\right)$ follows an exponential family form with additive causal influences from multiple parents, which may be restrictive in general cases. For instance, in brain imaging studies, fMRI data and EEG data capture different neural activities, and the interactions between brain regions are often highly nonlinear. Clearly, for general multimodal distributions (Figure 2), we cannot access the information redundancy assumed in Daunhawer et al. (2023); Von Kügelgen et al. (2021); Yao et al. (2023) and the nicely-behaved latent causal models in parametric assumptions (Morioka & Hyvarinen, 2023; 2024).

### Our high-level approach.

We divide the problem into two parts: we first identify latent subspaces ${\text{z}}^{\left(m\right)}$ (Section 4.1) and further disentangle identified subspaces into components $z_{i}^{\left(m\right)}$ (Section 4.2). For the subspace identification, we only assume that the information of the subspace ${\text{z}}^{\left(m\right)}$ is preserved in its corresponding observation ${\text{x}}^{\left(m\right)}$ and exerts sufficient influence on other modalities’ observations ${\text{x}}^{\left(-m\right)}$, thus weakening the redundancy assumption in previous work (Daunhawer et al., 2023; Yao et al., 2023). For the component-wise identification, we leverage a natural notion of structural sparsity in the literature (Zheng et al., 2022; Lachapelle et al., 2024) – the dependency among all the modalities should be explained with a minimal number of causal edges among latent subspaces ${\left\{{\text{z}}^{\left(m\right)}\right\}}_{m=1}^{M}$. This allows us to further disentangle each subspace into components, without resorting to parametric assumptions (Morioka & Hyvarinen, 2023; 2024).

### Notations.

We denote the dimensionality and the component indices of a given argument with d(⋅) and I(⋅), respectively. The notation −m represents the complement of modality m, while superscripts and subscripts enclosed in parentheses, such as (m), explicitly index modality m. We denote sub-matrices using the notation ${\left[\cdot \right]}_{R,C}$, where R and C are index sets corresponding to row and column selections, respectively. In this notation, setting R (or C) to : indicates the inclusion of all indices along the corresponding dimension.

### 4.1 Identifying Latent Subspaces

As previously discussed, we now provide the subspace identifiability. Formally, we would like to show that the estimated latent subspace ${\hat{\text{z}}}^{\left(m\right)}$ for any modality m and its true counterpart ${\text{z}}^{\left(m\right)}$ are equivalent up to an invertible map $h^{\left(m\right)}(\cdot )$, i.e., ${\hat{\text{z}}}^{\left(m\right)}=h^{\left(m\right)}\left({\text{z}}^{\left(m\right)}\right)$.

Given the data-generating process Eq. (2), the task is to remove modality-specific information $\eta^{\left(m\right)}$ from the observational data ${\text{x}}^{\left(m\right)}$ while retaining the latent variables ${\text{z}}^{\left(m\right)}$ causally related to other modalities. In light of this, we express the relations between the latent variables ${\text{z}}^{\left(m\right)}$ and the observation of its own modality ${\text{x}}^{\left(m\right)}$ and other modalities ${\text{x}}^{\left(-m\right)}$ as Eq. (3).

(3) $${\text{x}}^{\left(m\right)}=g_{{\text{x}}^{\left(m\right)}}\left({\text{z}}^{\left(m\right)},\eta^{\left(m\right)}\right),\;{\text{x}}^{\left(-m\right)}={\tilde{g}}_{{\text{x}}^{\left(-m\right)}}\left({\text{z}}^{\left(m\right)},{\tilde{\eta }}^{\left(-m\right)}\right),$$

where ${\tilde{\eta }}^{\left(-m\right)}$ denotes all the information necessary to generate the complement group ${\text{x}}^{\left(-m\right)}$ beyond ${\text{z}}^{\left(m\right)}$. ^2^ Consequently, ${\tilde{\eta }}^{\left(-m\right)}$ may admit causal/statistical relations with ${\text{z}}^{\left(m\right)}$. We denote the joint map of $g_{{\text{x}}^{\left(m\right)}}$ and ${\tilde{g}}_{{\text{x}}^{\left(-m\right)}}$ as ${\tilde{g}}^{\left(m\right)}:\left({\text{z}}^{\left(m\right)},\eta^{\left(m\right)},{\tilde{\eta }}^{\left(-m\right)}\right)\mapsto \text{x}$.

#### Condition 4.1 (Subspace Identifiability Conditions).

A1 [Smoothness & Invertibility]: The generating functions $g_{{\text{x}}^{\left(m\right)}}$ and ${\tilde{g}}^{\left(m\right)}$ are smooth and have smooth inverse functions.
A2 [Linear Independence]: The generating function ${\tilde{g}}_{{\text{x}}^{\left(-m\right)}}$ is smooth and its Jacobian columns corresponding to ${\text{z}}^{\left(m\right)}$ (i.e., ${\left[J_{{\tilde{g}}_{\text{x}}(-m)}\right]}_{:,I\left({\text{z}}^{\left(m\right)}\right)}$) are linearly independent almost anywhere.

#### Discussion on the conditions.

Condition 4.1-A1 requires that the information of the latent variables ${\text{z}}^{\left(m\right)}$ is preserved in its observation ${\text{x}}^{\left(m\right)}$, so that the identification of latent variables is well-defined (Hyvarinen et al., 2019; Khemakhem et al., 2020a; Von Kügelgen et al., 2021; Kong et al., 2023; Yao et al., 2023; Daunhawer et al., 2023). Since this holds for any modality m, the observations ${\text{x}}^{\left(-m\right)}$ should collectively preserve the information of other modalities ${\text{z}}^{\left(-m\right)}$.

Condition 4.1-A2 formalizes the notation of a minimal connectivity over modalities: ${\text{z}}^{\left(m\right)}$ should also exert sufficient influence on other modalities ${\text{z}}^{\left(-m\right)}$, so that the other modality observations ${\text{x}}^{\left(-m\right)}$ could be informative to identify ${\text{z}}^{\left(m\right)}$. This condition excludes degenerate scenarios where the causal influences between modalities are nearly negligible and is equivalent to local invertibility of ${\text{z}}^{\left(m\right)}$, which is strictly weaker than the global invertibility assumption in previous work (Daunhawer et al., 2023; Von Kügelgen et al., 2021; Yao et al., 2023) (e.g., $y=x^{2}$ is locally invertible but not globally so), as discussed earlier.

#### Theorem 4.2 (Subspace Identifiability).

*Let* $\theta :={\left\{g_{{\text{x}}^{\left(m\right)},},{\tilde{g}}_{\text{z}(-m)},p\left(\epsilon^{\left(m\right)}\right),p\left({\tilde{\epsilon }}^{\left(-m\right)}\right)\right\}}_{m=1}^{M}$ *and* $\hat{\theta }:={\left\{{\hat{g}}_{{\text{x}}^{\left(m\right)}},{\hat{\tilde{g}}}_{{\text{z}}^{\left(-m\right)}},p\left({\hat{\epsilon }}^{\left(m\right)}\right),p\left({\hat{\tilde{\epsilon }}}^{\left(-m\right)}\right)\right\}}_{m=1}^{M}$ *be two specifications of the data-generating process in* Eq. (3). *Suppose that they generate identical observational distributions (i.e.,* $p(\text{x})=\hat{p}(\text{x})$), θ *satisfies* Condition 4.1, *and* $\hat{\theta }$ *satisfies* Condition 4.1-A1. *The latent subspace* ${\hat{\text{z}}}^{\left(m\right)}$ *for any group m and its counterpart* ${\text{z}}^{\left(m\right)}$ *are equivalent up to an invertible map* $h^{\left(m\right)}(\cdot )$, *i.e*., ${\hat{\text{z}}}^{\left(m\right)}=h^{\left(m\right)}\left({\text{z}}^{\left(m\right)}\right)$.

#### Interpretation and proof sketch.

Theorem 4.2 states that one can disentangle the modality-specific information $\eta^{\left(m\right)}$ and the latent variables ${\text{z}}^{\left(m\right)}$ contained in the observation ${\text{x}}^{\left(m\right)}$ (which is a mixture of both). To achieve this, we leverage the fact that $\eta^{\left(m\right)}$ has no influence on other modalities ${\text{x}}^{\left(-m\right)}$, while ${\text{z}}^{\left(m\right)}$ has a non-trivial influence on ${\text{x}}^{\left(-m\right)}$, as characterized in Condition 4.1-A2. This crucial distinction provides sufficient footprints to disentangle these two subspaces for each modality, yielding the intended result.

### 4.2 Identifying Latent Components

Proceeding from the subspace identifiability (Theorem 4.2), we now further disentangle each subspace into individual components $z_{i}^{\left(m\right)}$ as outlined in Section 3. As foreshadowed, our key condition entails the sparsity of the graphical structures between modalities. Such dependency structures are captured in the generating function $g_{\text{z}}$ defined component-wise in Eq. (1), in particular its partial derivatives. We now introduce Condition 4.3, which facilitates component-wise identification.

#### Additional notations.

We denote the indices of the non-zero matrix entries by Supp(⋅). We denote the collection of partial derivatives among all latent components $\frac{\partial z_{i}^{\left(m\right)}}{\partial z_{j}^{\left(n\right)}}$ as a matrix function $G(\text{z},\epsilon )\in {\mathbb{R}}^{d(\text{z})\times d(\text{z})}$. We adopt diag(⋅) to denote matrices consisting of equally-sized square matrices on its diagonal and define T to possess the structure $T=\text{diag}\left(T_{1},\ldots ,T_{M}\right)$ with invertible $T_{m}\in {\mathbb{R}}^{d\left({\text{z}}^{\left(m\right)}\right)\times d\left({\text{z}}^{\left(m\right)}\right)}$. We denote the class of generalized permutation matrices of dimensionality d(z) as 𝓟(d(z)).

#### Condition 4.3 (Component Identifiability Conditions).

Over the domain of (z, ϵ), for any modality m and any T∉𝓟(d(z)), we have

(4) $$\sum_{m\neq n\in [M]}\|T_{m}^{-1}{\left[G\right]}_{\left(m),(n\right)}T_{n}{\|}_{0}>\sum_{m\neq n\in [M]}{\left\|{\left[G\right]}_{\left(m),(n\right)}\right\|}_{0}.$$

#### Discussion on the conditions.

**Figure 3:**
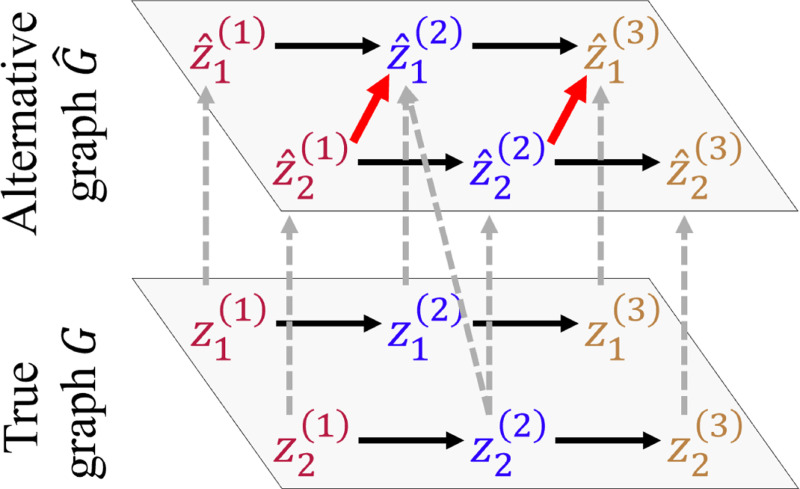
Denser graph $\hat{G}$.

Condition 4.3 stipulates sparse cross-modality causal connections among latent components z. Under this condition, if a latent component ${\hat{z}}_{i}^{\left(m\right)}$ is a function of two components ${\hat{z}}_{j}^{\left(m\right)}$ and ${\hat{z}}_{k}^{\left(m\right)}$ (when component-wise identification breaks down), the cross-modality causal connections in G are guaranteed to be denser than those in $\hat{G}$. We give a simple example to aid intuition in Figure 3: for a causal graph with three modalities, $z_{1}^{\left(1\right)}\to z_{1}^{\left(2\right)}\to z_{1}^{\left(3\right)}$ and $z_{2}^{\left(1\right)}\to z_{2}^{\left(2\right)}\to z_{2}^{\left(3\right)}$, suppose that ${\hat{z}}_{1}^{\left(2\right)}$ is a non-trivial mixture of $z_{1}^{\left(2\right)}$ and $z_{2}^{\left(2\right)}$ and other components are correctly identified, i.e., $\left[{\hat{z}}_{1}^{\left(1\right)},{\hat{z}}_{2}^{\left(1\right)},{\hat{z}}_{1}^{\left(2\right)},{\hat{z}}_{2}^{\left(2\right)},{\hat{z}}_{1}^{\left(3\right)},{\hat{z}}_{2}^{\left(3\right)}\right]=\left[z_{1}^{\left(1\right)},z_{2}^{\left(1\right)},h\left(z_{1}^{\left(2\right)},z_{2}^{\left(2\right)}\right),z_{2}^{\left(2\right)},z_{1}^{\left(3\right)},z_{2}^{\left(3\right)}\right]$. As a consequence, the alternative causal graph $\hat{G}$ would include additional edges ${\hat{z}}_{2}^{\left(1\right)}\to {\hat{z}}_{1}^{\left(2\right)}$ and ${\hat{z}}_{2}^{\left(2\right)}\to {\hat{z}}_{1}^{\left(3\right)}$, giving rise to a strictly denser graph. In Theorem 4.4, we show that this sparse structure could give us the desired component-wise identifiability under a proper sparse regularization constraint. The availability of multiple modalities greatly improves the feasibility of such sparsity conditions, especially with a large number of modalities, because the entanglement is limited within a single modality (owing to Theorem 4.1) and all other modalities can be leveraged to provide space for sparse connections.

Sparsity conditions have been embraced by the causal representation learning community (Lachapelle et al., 2024; Moran et al., 2022; Fumero et al., 2023; Xu et al., 2024). Especially relevant to our work is Zheng et al. (2022). As discussed above, we are obliged to deal with causal structures among all latent variables. In contrast, Zheng et al. (2022) assumes the sparsity of the causal connections between the latent variables and the observed variables – the directions (from the latent to the observed variables) are given and the children are directly observed. Notably, sparse properties manifest in biomedical systems of our interest, including gene regulatory networks (Milo et al., 2002; Babu et al., 2004; Nacher & Akutsu, 2013; Liu et al., 2011), metabolic systems (West et al., 2002;Banavar et al., 1999), and other living systems (Busiello et al., 2017), evidencing the plausibility of Condition 4.3 for biomedical applications.

#### Theorem 4.4 (Component-wise Identifiability).

*Let* $\theta :=\left({\left\{g_{{\text{x}}^{\left(m\right)}},g_{{\text{z}}^{\left(m\right)}},p\left(\epsilon^{\left(m\right)}\right)\right\}}_{m=1}^{M}\right)$ *and* $\hat{\theta }:=\left({\left\{{\hat{g}}_{{\text{x}}^{\left(m\right)}},{\hat{g}}_{{\text{z}}^{\left(m\right)}},\hat{p}\left(\epsilon^{\left(m\right)}\right)\right\}}_{m=1}^{M}\right)$ *be two specifications of the data-generating process in* Eq. (1) *and* Eq. (2). *Suppose that they generate identical observational distributions (i.e*., $p(\text{x})=\hat{p}(\text{x})$) *and* θ *satisfies* Condition 4.1 *and* Condition 4.3. *If* $\hat{\theta }$ *satisfies the following sparse regularization condition:*

(5) $$\sum_{m\neq n\in [M]}{\left\|{\left[\hat{G}\right]}_{\left(m),(n\right)}\right\|}_{0}\leq \sum_{m\neq n\in [M]}{\left\|{\left[G\right]}_{\left(m),(n\right)}\right\|}_{0},$$

*each component* $z_{i}^{\left(m\right)}$ *and its counterpart* ${\hat{z}}_{\pi (i)}^{\left(m\right)}$ *are equivalent up to an invertible map* h(⋅), *i.e*., ${\hat{z}}_{\pi (i)}^{\left(m\right)}=h\left(z_{i}^{\left(m\right)}\right)$ *under a permutation* π *over* $\left[d\left({\text{z}}^{\left(m\right)}\right)\right]$.

#### Interpretation and proof sketch.

The key idea of Theorem 4.4 is that for sparse causal graphs (as characterized in Condition 4.3), the mixing of latent components in any modality would introduce unnecessary causal edges connecting the other modalities. As the sparsity regularization Eq. (5) selects alternative models $\hat{\theta }$ that are not denser than the model θ, the mixing within each modality would be excluded. Consequently, each latent component ${\hat{z}}_{i}^{\left(m\right)}$ is a function of a unique component $z_{j}^{\left(m\right)}$, yielding the desired component-wise identifiability.

#### Implications.

In the context of biomedical applications, Theorem 4.4 indicates that under appropriate constraints, each component ${\hat{z}}_{i}^{\left(m\right)}$ in our estimation uniquely captures the information of an intrinsic biomedical factor behind the medical measurements (e.g., genetic predisposition). Therefore, the learned representation enjoys strong interpretability under theoretical guarantees, which is often lacking in existing biomedical models, as noted in Section 2. Theorem 4.2 and Theorem 4.4 provide insights for practical model design, which we employ in our architecture in Section 5.

#### Shared latent variables.

Certain applications may involve latent variables that are shared across modalities. In such scenarios, we can employ contrastive learning objectives and the associated theoretical guarantees in previous work (Yao et al., 2023; Daunhawer et al., 2023; Von Kügelgen et al., 2021) as a pre-processing procedure and treat such shared latent variables as separate modalities in our implementation. Please refer to Appendix C.3, C.4, and E for detailed discussion and results.

## 5 Estimation Model Architectures

**Figure 4:**
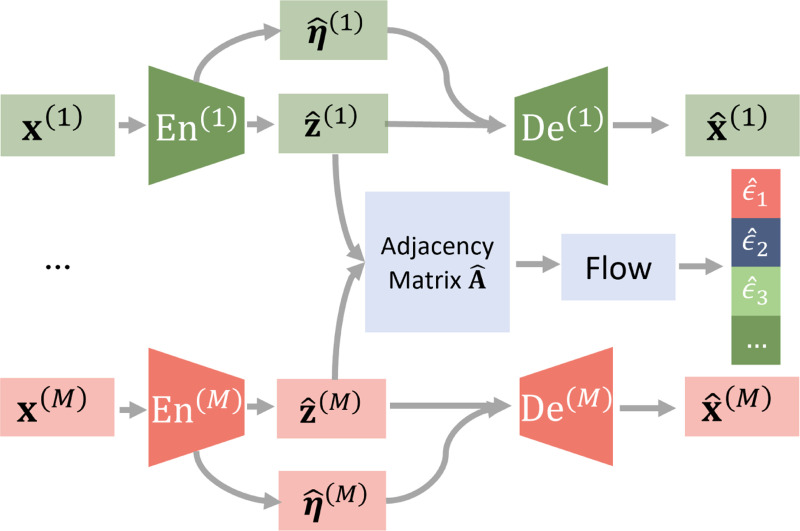
Estimation framework. Given multi-modal observations $\left({\text{x}}^{\left(1\right)},\ldots ,{\text{x}}^{\left(M\right)}\right)$, the latent variables and domain-specific information in modality m are inferred as ${\hat{\text{z}}}^{\left(m\right)}$ and ${\hat{\eta }}^{\left(m\right)}$ by individual encoders. The observations are then reconstructed with corresponding decoders as ${\hat{\text{x}}}^{\left(m\right)}$. We enforce independence conditions by minimizing the KL divergence term $D_{\text{KL}}\left(\left[{\left\{{\hat{\eta }}^{\left(m\right)}\right\}}_{m=1}^{M},{\left\{{\hat{\epsilon }}_{i}\right\}}_{i=1}^{d(\text{z})}\right]∥𝓝(0,I)\right)$. We enforce the sparsity constraint by minimizing the ${𝓛}_{1}$ norm in the inferred adjacency matrix $\hat{\text{A}}$.

Given the identifiability results, we further propose an estimation framework (shown in Figure 4) that enforces the proposed assumptions as constraints to identify the latent variables in each modality.

### Encoder and decoder.

Each modality ${\text{x}}^{\left(m\right)}$ is given as an input to the corresponding encoder and outputs the estimated latent ${\hat{\text{z}}}^{\left(m\right)}$ and domain-specific information ${\hat{\eta }}^{\left(m\right)}$. They are then concatenated and passed to the corresponding decoder to reconstruct the observations as ${\hat{\text{x}}}^{\left(m\right)}$. The reconstruction loss is calculated using the mean squared error (MSE) as ${𝓛}_{\text{Recon }}={\sum_{m=1}^{M}\left\|x^{\left(m\right)}-{\hat{x}}^{\left(m\right)}\right\|}_{2}^{2}$.

### Conditional independence constraints.

Given Eq (3), we enforce the conditional independence condition ${\text{x}}^{\left(m\right)}⫫{\text{x}}^{\left(n\right)}|{\text{z}}^{\left(m\right)}$ and the independence condition on $\eta^{\left(m\right)}⫫{\text{z}}^{\left(m\right)}$ by enforcing independence among components in $\gamma =\left[{\left\{{\hat{\eta }}^{\left(m\right)}\right\}}_{m=1}^{M},{\left\{{\hat{\epsilon }}_{i}\right\}}_{i=1}^{d(\text{z})}\right]$. Such equivalence is shown in Propositions 5.1 and 5.2, and proofs are provided in Appendix B. Specifically, we minimize the KL divergence loss between the posterior and a Gaussian prior distribution: ${𝓛}_{\text{Ind}}=D_{\text{KL}}(p(\gamma )∥𝓝(0,I))$.

### Proposition 5.1. *[Conditional Independence Condition]*

*Let* ${\text{x}}^{\left(m\right)}$ *and* ${\text{x}}^{\left(n\right)}$ *be two different multi-modal observations*. ${\text{z}}^{\left(m\right)}\subset \text{z}$ *are the set of block-identifying latent variables, and* $\eta^{\left(m\right)}\subset \eta$ *are domain-specific information in modality* m. *We have* ${\text{x}}^{\left(m\right)}⊥{\text{x}}^{\left(n\right)}|{\text{z}}^{\left(m\right)}\Leftrightarrow \eta^{\left(m\right)}⊥\eta^{\left(n\right)}$.

### Proposition 5.2. *[Independent Noise Condition]*

*Let* z *and* η *be the block-identified latent variables and domain-specific information, respectively, across all modalities. Denote* ϵ *as the exogenous variables in the latent causal structure. We have* η⊥z⇔η⫫ϵ.

### Sparsity regularization.

We use flow to estimate the exogenous variables ϵ in Eq. (1) and implement the causal relations through a learnable adjacency matrix $\hat{\text{A}}$. The binary values in $\hat{\text{A}}$ represent the causal generation process between latent variables, e.g. ${\hat{A}}_{i,j}=1$ indicates ${\hat{z}}_{j}$ is the parent of ${\hat{z}}_{i}$, while ${\hat{A}}_{i,j}=0$ means ${\hat{z}}_{j}$ dose not contribute to the generation of ${\hat{z}}_{i}$. For each component ${\hat{z}}_{i}$, we select its parents $\text{Pa}\left({\hat{z}}_{i}\right)$ based on the adjacency matrix, and apply the flow transformation to get ${\hat{\epsilon }}_{i}$.

To encourage sparsity among the latent variables $\hat{\text{z}}$, we impose a regularization term on the learned adjacency matrix. Based on the sparsity assumption, the optimal causal graph should be the minimal one that still allows the model to accurately match the ground truth generative distribution. To achieve this, we reduce the dependencies between different components of $\hat{\text{z}}$ by adding a ${𝓛}_{1}$ penalty on the adjacency matrix, s.t., ${𝓛}_{\text{Sp}}=∥\hat{\text{A}}{∥}_{1}$.

### Optimization.

The model parameters are optimized using the combination objective:

(6) $$𝓛=\alpha_{\text{Recon}}{𝓛}_{\text{Recon}}+\alpha_{\text{Ind}}{𝓛}_{\text{Ind}}+\alpha_{\text{Sp}}{𝓛}_{\text{Sp}}.$$

## 6 Experiment Results

To evaluate the efficacy of our proposed method, we conduct extensive experiments on (1) numerical, (2) synthetic and (3) real-world datasets. In terms of the baselines, we compare our method with: (1) BetaVAE (Higgins et al., 2017), which does not consider causal relations in the latent space. (2) CausalVAE (Yang et al., 2020), which considers the causally related latent variables with a single modality. (3) Multimodal contrastive learning (MCL) (Daunhawer et al., 2023), which recovers the shared latent factors from multiple modalities. Throughout the experiments, we consider the following evaluation metrics: (1) Mean Correlation Coefficient (MCC) measures how well the estimated latent variables match the true ones, with an MCC of 1 indicating perfect identifiability up to component-wise transformations. (2) R2 measures the proportion of variance in the ground truth latent that is explained by the estimated latent, with a value of 1 indicating that all variance is explained. (3) Structural Hamming Distance (SHD) compares graphs by their adjacency matrices, where a lower SHD indicates stronger similarity between graphs.

### 6.1 Numerical Dataset

#### Setup.

In the numerical simulations, we consider three cases with different numbers of modalities and inter-modal causal relations. Case 1: 15-dimensional observations across two modalities, each with two latent and one exogenous variable. Case 2: 20-dimensional observations across two modalities, each with three latent and one exogenous variable. Case 3: 30-dimensional observations across four modalities, each with two latent and one exogenous variable. The nonparametric mixing function is simulated by a random MLP with LeakyReLU units, and the inter-modal latent variables are sparse causally related. The detailed data-generation process is provided in Appendix D.1.

#### Results and ablation.

**Figure 5:**
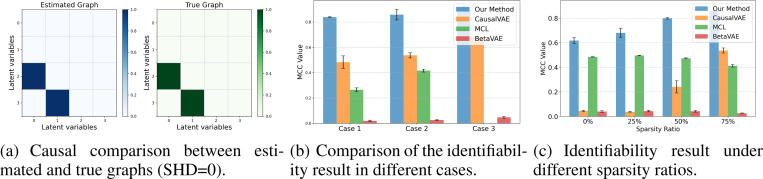
Numerical experiment results. (a) Successful recovery of the inter-modal causal graph. (b) Baseline comparisons in different cases. (c) Result of sparsity ablation study.

Figure 5 shows the identifiability results in different cases, where the high MCC indicates the successful recovery of the latent variables. The inter-modal causal relations are successfully recovered (SHD=0) and the causal comparison result in case 1 is shown in Figure 5(a). The identifiability comparison results are shown in Figure 5(b) (MCL is not applicable in case 3 due to the two-modality constraint). CausalVAE requires additional supervision signals to establish identifiability, and MCL assumes content invariance and can only block-identify latent variables. In general, these baselines neither account for the multimodal setting nor the modality-specific latent variables, and therefore do not recover the latent variables.

As an ablation study, we further show the consequences of violating the sparsity assumptions to validate our theorem. Based on case 2, we create four types of datasets with different sparsity ratios and report the MCC in each scenario in Figure 5(c). The sparsity ratio represents the ratio of existing causal links to all possible causal links between modality-specific latent variables. A value of 0 indicates that the latent variables between modalities are fully connected, while higher values correspond to sparser connections. The result shows that identifiability can be better achieved with a higher sparsity ratio, and our framework outperforms other baselines in all scenarios.

### 6.2 Synthetic Dataset: Variant Mnist

#### Setup.

We manually create a variant of the MNIST dataset to encode causal relationships between different modalities, using colored MNIST (Arjovsky et al., 2019) and fashion MNIST (Xiao et al., 2017) as two different modalities. In colored MNIST, the horizontal position of the digit influences the image transparency. This horizontal position further serves as a causal factor for the vertical position of the fashion items in the fashion MNIST, which influences image grayscale. This design ensures a structured causal dependency across modalities while maintaining a non-deterministic mapping. Further data descriptions are provided in the Appendix D.2.

#### Results.

Table 2 presents the results of the identifiability comparison, where higher MCC and R2 indicate better performance of our method. BetaVAE does not explicitly model causal relationships among latent variables, leading to suboptimal recovery in our setting. CausalVAE, which relies on additional supervision, fails to recover the latent variables effectively.

### 6.3 Real-World Dataset: Human Phenotype

The human phenotype dataset (Shilo et al., 2021) is a large-scale, longitudinal collection of phenotypic profiles from a diverse global population. It includes comprehensive human health data and provides a comprehensive view of health and disease factors. The dataset contains various types of participant information, categorized into tabular, time series, and image data. Specifically, it includes health information across 30 modalities, such as blood tests, anthropometry, fundus imaging, etc. Detailed data descriptions can be found in the Appendix D.3.

**Figure 6:**
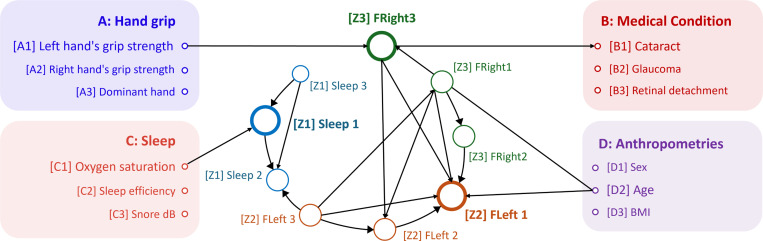
Causal analysis results across different modalities, including hand grip, medical conditions, sleep, and anthropometrics. We ran the causal algorithm on all variables, but for clarity only report the causal relationships that have direct connections to the estimated latent variables.

In this work, we focus on the time-series sleep monitoring dataset (*Sleep*) and the fundus imaging dataset for both left and right eyes (*FLeft* and *FRight*) to estimate the latent factors (Z1, Z2, Z3) underlying each modality. We applied the PC algorithm (Spirtes et al., 2001) to discover causal relationships between the estimated latent variables and other four additional tabular modalities (A, B, C, D), providing an implicit evaluation on the effectiveness. The result with direct causal relations is shown in Figure 6, where variables from the same modality have the same color and different modalities have different colors.

A key finding is that the discovered causal relationships are consistent with findings from medical research. For example, *Sleep 1* shows a direct causal relationship with *Oxygen saturation*, suggesting that sleep conditions may influence blood oxygen levels. This observation is consistent with previous studies (Wali et al., 2020). In addition, the fundus-related latent variables *FRight 1* and *FLeft 1* have a direct causal relationship with *Age*, suggesting that aging plays an important role in changes in retinal health (Ege et al., 2002; Einbock et al., 2005). Interestingly, the fundus image of the right eye has a direct causal relationship with the grip strength of the left hand, as recently demonstrated in biomedical research (Bikbov et al., 2023; Qiu et al., 2020).

## 7 Conclusion and Limitations

In this work, we develop a theoretically grounded framework for recovering latent causal variables from multi-modal observations. Extensive experimental results on synthetic and real-world datasets demonstrate the practical effectiveness of our approach.

### Limitations:

Empirically, our framework assumes prior knowledge of the number of latent variables in each modality, which may be unrealistic in real-world scenarios. Additionally, a detailed evaluation against the quantitative benchmarks used in biomedical models remains an area for future exploration.

## Supplementary Material

Supplement 1

## Figures and Tables

**Table 1:** Related work on multimodal causal representation learning. This table considers whether a method can accommodate more than two modalities, whether the latent variable distribution is nonparametric, whether it allows dependency among latent variables, and whether identifiability is component-wise.

Related work	> 2 Modalities	Nonparam. Dist.	Latent Dependency	Component-wise Iden.

Gresele et al. (2020)	✓	×	×	✓
Von Kügelgen et al. (2021)	×	✓	✓	×
Daunhawer et al. (2023)	×	✓	✓	×
Yao et al. (2023)	✓	✓	✓	×
Morioka & Hyvarinen (2024)	✓	×	✓	✓
**Our work**	✓	✓	✓	✓

**Table 2:** The results of MNIST dataset.

	MCL	BetaVAE	CausalVAE	Ours
R2	0.79 ± 6e-5	0.68 ± 2e-3	0.50 ± 4e-3	**0.90** ± 9e-5
MCC	0.63 ± 2e-6	0.53 ± 1e-3	0.74 ± 2e-3	**0.85** ± 3e-5

## Footnotes

1 Please see Appendix A for details on the notion of identifiability.

2 We use $\tilde{・}$ to differentiate ${\tilde{\eta }}^{\left(-m\right)}$ from a collection of modality-specific variables η defined in Eq. (2).
