# Supplementary material for: Causal Representation Learning from Multi-modal Biomedical Observations
Source: ArXiv. 2025 Mar 16:arXiv:2411.06518v3. Preprint. [Version 3] (PMC11952583)
Supplement: Supplement 1 [file NIHPP2411.06518v3-supplement-1.pdf]

Supplementary Materials for "Causal Representation Learning from Multimodal Biomedical Observations"

CONTENTS

A Notation and Terminology **18**

B Constraints in the Estimation Framework **18**

C Identifiability Theory **20**

C.1 Proof for Theorem 4.2 20

C.2 Proof for Theorem 4.4 21

C.3 Extended Theorem 4.2 and its Proof 23

C.4 Extended Theorem 4.4 and its Proof 24

D Experimental Details **27**

D.1 Numerical Dataset 27

D.2 Synthetic Dataset 27

D.3 Real-world Dataset 28

D.4 Evaluation Metrics 28

D.5 Detailed Discussion on Human Phenotype 29

E Extended Experiment **29**

F Implementation Details **30**

F.1 Network Architecture 31

F.2 Training Details 31

G Algorithm Pseudocode **31**

A Notation and Terminology

We summarize the notations used throughout the paper in Table 3.

**Index**	
m, n	Modality index
i, j	Variable element index
$I(\cdot)$	Component indices of a given argument
$d(\cdot)$	Dimensionality indices of a given argument
**Variable**	
$\mathbf{x}^{(m)}$	Observation/measurement in each modality
$\mathbf{z}^{(m)}$	Causally related latent variables in each modality
$\mathbf{x}^{(m)}, \mathbf{x}^{(-m)}$	One specific observation in modality m, and the rest of others
$\mathbf{z}^{(m)}, \mathbf{z}^{(-m)}$	One specific latent variables in modality m, and the rest of others
η	Domain-specific information
ϵ	Mutually independent exogenous variables
$\hat{z}_i$	Estimated latent variables over z_i
$\hat{\mathbf{x}}^{(m)}$	Reconstructed observation in modality m
$\text{Pa}(z_i^{(m)})$	Set of direct cause nodes/parents of variable $z_i^{(m)}$
**Function and Hyperparameter**	
$g_{z_i^m}$	Causal function among latent variables
$g_{\mathbf{x}^{(m)}}$	Nonparametric mixing function in modality m
h	Invertible mapping from true latent to the estimated latent
p	Distribution function (e.g., p_{z_i} is the distribution of z_i)
$\alpha_{\text{Recon}}, \alpha_{\text{Ind}}, \alpha_{\text{Sp}}$	Weights in the combination objective

Table 3: List of notations.

Notions of identifiability. Following the literature on ICA (Hyvarinen & Morioka, 2016; Hyvarinen et al., 2019; Comon, 1994) and causal representation learning (Yao et al., 2023; Von Kügelgen et al., 2021; Daunhawer et al., 2023), we assume that the generating function $g_{\mathbf{x}^{(m)}}$ (Eq. (2)) is an invertible map from $(\mathbf{z}^{(m)}, \eta^{(m)})$ to $\mathbf{x}^{(m)}$ (Condition 4.1). Under this invertibility assumption, given the value of $\mathbf{x}^{(m)}$, one can perfectly determine the value of $\mathbf{z}^{(m)}$, which is essentially the posterior $p(\mathbf{z}|\mathbf{x})$ (a point mass here). Here, $\mathbf{z}^{(m)}$ is a function of $\mathbf{x}^{(m)}$, and the identifiability of g gives rise to the result that $\mathbf{z}^{(m)}$ values can be identified from $\mathbf{x}^{(m)}$.

In statistics, to show identifiability, we start with equal distributions $p_{\phi_1} = p_{\phi_2}$ to derive the equivalence of the parameters $\phi_1 = \phi_2$. In our case, since the functions $g_{\mathbf{x}^{(m)}}, \hat{g}_{\mathbf{x}^{(m)}}$ are invertible, one can reason about the relation between the two specifications $g_{\mathbf{x}^{(m)}}$ and $\hat{g}_{\mathbf{x}^{(m)}}$ through a composition $h := \hat{g}^{-1}_{\mathbf{x}^{(m)}} \circ g_{\mathbf{x}^{(m)}}$. For instance, h is the identity when the two specifications are identical. Similarly, in this work, we start with equal values of $\mathbf{x}$ to establish the relation between $\mathbf{z}$ and $\hat{\mathbf{z}}$.

B Constraints in the Estimation Framework

Here we provide the proofs for the constraints utilized in the estimation framework.

Proposition B.1. *[Conditional Independence Condition] Let $\mathbf{x}^{(m)}$ and $\mathbf{x}^{(n)}$ be two different multi-modal observations. $\mathbf{z}^{(m)} \subset \mathbf{z}$ are the set of block-identifying latent variables, and $\eta^{(m)} \subset \eta$ are domain-specific information in modality m. We have*

$$\mathbf{x}^{(m)} \perp\!\!\!\perp \mathbf{x}^{(n)} \,|\, \mathbf{z}^{(m)} \Longleftrightarrow \eta^{(m)} \perp\!\!\!\perp \eta^{(n)}. \tag{7}$$

Proof. Given the data generation process in Eq. (2), the following assumptions hold true for any $m, n \in [M]$: (1) $\mathbf{z}^{(m)} \perp\!\!\!\perp \eta^{(m)}$; (2) $\mathbf{z}^{(m)} \perp\!\!\!\perp \eta^{(n)}$; (3) $\eta^{(m)} \perp\!\!\!\perp \mathbf{x}^{(n)}$.

Sufficient condition. Given LHS of Eq. (7), we have

$$p(\mathbf{x}^{(m)}, \mathbf{x}^{(n)} \mid \mathbf{z}^{(m)}) = p(\mathbf{x}^{(m)} \mid \mathbf{z}^{(m)}) p(\mathbf{x}^{(n)} \mid \mathbf{z}^{(m)}).$$

$$\begin{aligned}
\xrightarrow{RHS} p(\mathbf{x}^{(m)}, \mathbf{x}^{(n)} \mid \mathbf{z}^{(m)}) &= \frac{p(\mathbf{x}^{(m)}, \mathbf{x}^{(n)}, \mathbf{z}^{(m)})}{p(\mathbf{z}^{(m)})} = \frac{p(\eta^{(m)}, \eta^{(n)}, \mathbf{z}^{(m)})}{p(\mathbf{z}^{(m)})} |\text{det} \frac{\partial \eta^{(m)}}{\partial \mathbf{x}^{(m)}}||\text{det} \frac{\partial \eta^{(n)}}{\partial \mathbf{x}^{(n)}}| \\
&= p(\eta^{(m)}, \eta^{(n)} \mid \mathbf{z}^{(m)}) |\text{det} \frac{\partial \eta^{(m)}}{\partial \mathbf{x}^{(m)}}||\text{det} \frac{\partial \eta^{(n)}}{\partial \mathbf{x}^{(n)}}| \\
\xrightarrow{LHS} p(\mathbf{x}^{(m)} \mid \mathbf{z}^{(m)}) p(\mathbf{x}^{(n)} \mid \mathbf{z}^{(m)}) &= \frac{p(\mathbf{x}^{(m)}, \mathbf{z}^{(m)})}{p(\mathbf{z}^{(m)})} \frac{p(\mathbf{x}^{(n)}, \mathbf{z}^{(m)})}{p(\mathbf{z}^{(m)})} \\
&= \frac{p(\eta^{(m)}, \mathbf{z}^{(m)})}{p(\mathbf{z}^{(m)})} |\text{det} \frac{\partial \eta^{(m)}}{\partial \mathbf{x}^{(m)}}| \frac{p(\eta^{(n)}, \mathbf{z}^{(m)})}{p(\mathbf{z}^{(m)})} |\text{det} \frac{\partial \eta^{(n)}}{\partial \mathbf{x}^{(n)}}| \\
&= p(\eta^{(m)} \mid \mathbf{z}^{(m)}) p(\eta^{(n)} \mid \mathbf{z}^{(n)}) |\text{det} \frac{\partial \eta^{(m)}}{\partial \mathbf{x}^{(m)}}||\text{det} \frac{\partial \eta^{(n)}}{\partial \mathbf{x}^{(n)}}|
\end{aligned}$$

Thus we have

$$p(\eta^{(m)}, \eta^{(n)} | \mathbf{z}^{(m)}) = p(\eta^{(m)} | \mathbf{z}^{(m)}) p(\eta^{(n)} | \mathbf{z}^{(n)}) \Rightarrow p(\eta^{(m)}, \eta^{(n)}) = p(\eta^{(m)}) p(\eta^{(n)}) \Rightarrow \eta^{(m)} \perp\!\!\!\perp \eta^{(n)}$$

Necessary condition. Given RHS of Eq. (7) and above conclusion, we have

$$p(\mathbf{x}^{(m)} \mid \mathbf{z}^{(m)}) = p(\eta^{(m)}) |\text{det} \frac{\partial \eta^{(m)}}{\partial \mathbf{x}^{(m)}}|, \quad p(\mathbf{x}^{(n)} \mid \mathbf{z}^{(m)}) = p(\eta^{(n)}) |\text{det} \frac{\partial \eta^{(n)}}{\partial \mathbf{x}^{(n)}}|$$

$$\begin{aligned}
&\xrightarrow{Multiplication} p(\mathbf{x}^{(m)} \mid \mathbf{z}^{(m)}) p(\mathbf{x}^{(n)} \mid \mathbf{z}^{(m)}) = p(\eta^{(m)}) p(\eta^{(n)}) |\text{det} \frac{\partial \eta^{(m)}}{\partial \mathbf{x}^{(m)}}||\text{det} \frac{\partial \eta^{(n)}}{\partial \mathbf{x}^{(n)}}| \\
&= p(\eta^{(m)}, \eta^{(n)}) |\text{det} \frac{\partial \eta^{(m)}}{\partial \mathbf{x}^{(m)}}||\text{det} \frac{\partial \eta^{(n)}}{\partial \mathbf{x}^{(n)}}| = \frac{p(\eta^{(m)}, \eta^{(n)}, \mathbf{z}^{(m)})}{p(\mathbf{z}^{(m)})} |\text{det} \frac{\partial \eta^{(m)}}{\partial \mathbf{x}^{(m)}}||\text{det} \frac{\partial \eta^{(n)}}{\partial \mathbf{x}^{(n)}}| = \frac{p(\mathbf{x}^{(m)}, \mathbf{x}^{(n)}, \mathbf{z}^{(m)})}{p(\mathbf{z}^{(m)})} \\
&\Rightarrow p(\mathbf{x}^{(m)} \mid \mathbf{z}^{(m)}) p(\mathbf{x}^{(n)} \mid \mathbf{z}^{(m)}) = p(\mathbf{x}^{(m)}, \mathbf{x}^{(n)} \mid \mathbf{z}^{(m)}) \Rightarrow \mathbf{x}^{(m)} \perp\!\!\!\perp \mathbf{x}^{(n)} \mid \mathbf{z}^{(m)}
\end{aligned} \tag{8}$$

□

Proposition B.2 (Independent Noise Condition). *Let* $\mathbf{z}$ *and* η *be the block-identified latent variables and domain-specific information, respectively, across all modalities. Denote* ϵ *as the exogenous variables in the latent causal structure. We have*

$$\eta \perp\!\!\!\perp \mathbf{z} \Longleftrightarrow \eta \perp\!\!\!\perp \epsilon. \tag{9}$$

Proof. Given the causal function in Eq. (1), we have $p(\mathbf{z}) = p(\epsilon)|\text{det}\frac{\partial \epsilon}{\partial \mathbf{z}}|$.

Sufficient condition. Suppose $(\mathbf{z}, \eta) = h(\epsilon, \eta)$ and $\eta \perp\!\!\!\perp \mathbf{z}$, we have

$$\begin{aligned}
p(\mathbf{z}, \eta) = p(\epsilon, \eta) |\text{def} \frac{\partial \epsilon}{\partial \mathbf{z}}| \Rightarrow p(\mathbf{z}) p(\eta) = p(\epsilon, \eta) |\text{def} \frac{\partial \epsilon}{\partial \mathbf{z}}| \Rightarrow p(\epsilon) p(\eta) |\text{det} \frac{\partial \epsilon}{\partial \mathbf{z}}| = p(\epsilon, \eta) |\text{def} \frac{\partial \epsilon}{\partial \mathbf{z}}| \\
\Rightarrow p(\epsilon) p(\eta) = p(\epsilon, \eta) \Rightarrow \eta \perp\!\!\!\perp \epsilon
\end{aligned} \tag{10}$$

Necessary condition. Suppose $(\mathbf{z}, \eta) = h(\epsilon, \eta)$ and $\eta \perp\!\!\!\perp \epsilon$, we have

$$p(\mathbf{z}, \eta) = p(\epsilon, \eta) |\text{def} \frac{\partial \epsilon}{\partial \mathbf{z}}| \Rightarrow p(\mathbf{z}, \eta) = p(\epsilon) |\text{def} \frac{\partial \epsilon}{\partial \mathbf{z}}| p(\eta) \Rightarrow p(\mathbf{z}, \eta) = p(\mathbf{z}) p(\eta) \Rightarrow \eta \perp\!\!\!\perp \mathbf{z} \tag{11}$$

□

C IDENTIFIABILITY THEORY

C.1 PROOF FOR THEOREM 4.2

We present the proof for Theorem 4.2. For ease of reference, we duplicate Condition 4.1 and Theorem 4.2 below.

Condition 4.1 (Subspace Identifiability Conditions)**.**

A1 [Smoothness & Invertibility]: The generating functions $g_{\mathbf{x}^{(m)}}$ and $\tilde{g}^{(m)}$ are smooth and have smooth inverse functions.

A2 [Linear Independence]: The generating function $\tilde{g}_{\mathbf{x}^{(-m)}}$ is smooth and its Jacobian columns corresponding to $\mathbf{z}^{(m)}$ (i.e., $[\boldsymbol{J}_{\tilde{g}_{\mathbf{x}^{(-m)}}}]_{:,I(\mathbf{z}^{(m)})}$) are linearly independent almost anywhere.

Theorem 4.2 (Subspace Identifiability)**.** *Let* $\boldsymbol{\theta} := \{g_{\mathbf{x}^{(m)}}, \tilde{g}_{\mathbf{z}^{(-m)}}, p(\boldsymbol{\epsilon}^{(m)}), p(\tilde{\boldsymbol{\epsilon}}^{(-m)})\}_{m=1}^{M}$ *and* $\hat{\boldsymbol{\theta}} := \{\hat{g}_{\mathbf{x}^{(m)}}, \hat{\tilde{g}}_{\mathbf{z}^{(-m)}}, p(\hat{\boldsymbol{\epsilon}}^{(m)}), p(\hat{\tilde{\boldsymbol{\epsilon}}}^{(-m)})\}_{m=1}^{M}$ *be two specifications of the data-generating process in Eq. (3). Suppose that they generate identical observational distributions (i.e.,* $p(\mathbf{x}) = \hat{p}(\mathbf{x})$*),* $\boldsymbol{\theta}$ *satisfies Condition 4.1, and* $\hat{\boldsymbol{\theta}}$ *satisfies Condition 4.1-A1. The latent subspace* $\hat{\mathbf{z}}^{(m)}$ *for any group* m *and its counterpart* $\mathbf{z}^{(m)}$ *are equivalent up to an invertible map* $h^{(m)}(\cdot)$*, i.e.,* $\hat{\mathbf{z}}^{(m)} = h^{(m)}(\mathbf{z}^{(m)})$*.*

Proof. Given the generating processes in Eq. (2) and Eq. (1), we can express any observed group $\mathbf{x}^{(m)}$ and its complement $\mathbf{x}^{(-m)} := \mathbf{x} \setminus \mathbf{x}^{(m)}$ as two views of the latent variables of group m:

$$\mathbf{x}^{(m)} := g^{(m)}(\mathbf{z}^{(m)}, \boldsymbol{\eta}^{(m)}), \tag{12}$$

$$\mathbf{x}^{(-m)} := g^{(-m)}(\mathbf{z}^{(m)}, \tilde{\boldsymbol{\eta}}^{(-m)}), , \tag{13}$$

where $\boldsymbol{\eta}^{(m)}$ stands for exogenous variables for the group $\mathbf{x}^{(m)}$ and $\tilde{\boldsymbol{\eta}}^{(-m)}$ represents all the information necessary to generate the complement group $\mathbf{x}^{(-m)}$ beyond $\mathbf{z}^{(m)}$.

Following the classic definition of identifiability, we define two specifications $\boldsymbol{\theta} = \{g_{\mathbf{x}^{(m)}}, g_{\mathbf{z}^{(m)}}, p(\boldsymbol{\epsilon}^{(m)})\}_{m=1}^{M}$ and $\hat{\boldsymbol{\theta}} := \{\hat{g}_{\mathbf{x}^{(m)}}, \hat{g}_{\mathbf{z}^{(m)}}, \hat{p}(\boldsymbol{\epsilon}^{(m)})\}_{m=1}^{M}$ that fit the observation distribution $p(\mathbf{x})$. To show the identifiability in terms of the functions in $\boldsymbol{\theta}$ and $\hat{\boldsymbol{\theta}}$, we show that given the same $\mathbf{x}^{(m)}$ value the identifiability between $\mathbf{z}^{(m)}$ and $\hat{\mathbf{z}}^{(m)}$.

Thus, the subspace identification is equivalent to show that for each group m, the estimated latent variable $\hat{\mathbf{z}}^{(m)}$ and the true counterpart are related via an invertible map h, i.e., $\hat{\mathbf{z}}^{(m)} = h(\mathbf{z}^{(m)})$.

Eq. (12) and the invertibility of the map $(\mathbf{z}, \boldsymbol{\eta}^{(m)}, \tilde{\boldsymbol{\eta}}^{(-m)}) \mapsto (\mathbf{x}^{(m)}, \mathbf{x}^{(-m)})$ (Condition 4.1-A1) give rise to an invertible map $\tilde{h} : (\hat{\mathbf{z}}^{(m)}, \hat{\boldsymbol{\eta}}^{(m)}, \hat{\tilde{\boldsymbol{\eta}}}^{(-m)}) \mapsto (\mathbf{z}^{(m)}, \boldsymbol{\eta}^{m}, \tilde{\boldsymbol{\eta}}^{(-m)})$.

The matched observed distribution between the true and the estimated models for the generating process Eq. (13) yields that

$$g^{(-m)}(\mathbf{z}^{(m)}, \tilde{\boldsymbol{\eta}}^{(-m)}) = \hat{g}^{(-m)}(\hat{\mathbf{z}}^{(m)}, \hat{\tilde{\boldsymbol{\eta}}}^{(-m)}). \tag{14}$$

Plugging in $\tilde{h}$ gives

$$\hat{g}^{(-m)}(\hat{\mathbf{z}}^{(m)}, \hat{\tilde{\boldsymbol{\eta}}}^{(-m)}) = g^{(-m)}\left(\left[\tilde{h}\left(\hat{\mathbf{z}}^{(m)}, \hat{\boldsymbol{\eta}}^{(m)}, \hat{\tilde{\boldsymbol{\eta}}}^{(-m)}\right)\right]_{I(\mathbf{z}^{(m)}), I(\tilde{\boldsymbol{\eta}}^{(-m)})}\right). \tag{15}$$

where we adopt $I(\cdot)$ to indicate the indices of its argument.

For any $i \in [d(\mathbf{x}^{(m)})]$ and $j \in [d(\hat{\boldsymbol{\eta}}^{(m)})]$, we take partial derivative w.r.t. $\hat{\eta}_j^{(m)}$ on both sides of Eq. (15):

$$\underbrace{\frac{\partial[\hat{g}^{(-m)}]_i}{\partial[\hat{\eta}^{(m)}]_j}}_{=0} = \frac{\partial[g^{(-m)}]_i}{\partial[\hat{\eta}^{(m)}]_j}. \tag{16}$$

The left-hand side of Eq. (15) equals to zero because $\hat{g}^{(-m)}$ is not a function of $\hat{\boldsymbol{\eta}}^{(m)}$.

Therefore, expanding the right-hand side of Eq. (15) gives:

$$\sum_{k\in I(\mathbf{z}^{(-m)})\cup I(\tilde{\boldsymbol{\eta}}^{(-m)})} \frac{\partial [g^{(-m)}]_i}{\partial [\tilde{h}]_k} \cdot \frac{\partial [\tilde{h}]_k}{\partial [\hat{\eta}^{(m)}]_j} = \sum_{k\in I(\mathbf{z}^{(-m)})} \frac{\partial [g^{(-m)}]_i}{\partial [\tilde{h}]_k} \cdot \frac{\partial [\tilde{h}]_k}{\partial [\hat{\eta}^{(m)}]_j} = 0. \tag{17}$$

The first equality in Eq. (17) is due to the fact that $\tilde{\boldsymbol{\eta}}^{(-m)}$ is a function of $\mathbf{x}^{(-m)}$ and varying $\hat{\boldsymbol{\eta}}^{(m)}$ doesn't vary $\mathbf{x}^{(-m)}$ ($\hat{\boldsymbol{\eta}}^{(m)}$ is a function of $\mathbf{x}^{(m)}$ thanks to the invertibility of $\hat{g}^{(m)}$), i.e., $\frac{\partial [\tilde{\eta}^{(-m)}]_k}{\partial [\hat{\eta}^{(m)}]_j} = 0$.

Condition 4.1-A2 implies that the matrix $\left(\frac{\partial [g^{(-m)}]_i}{\partial [\tilde{h}]_k}\right)_{i,k}$ has a full column rank. Therefore, its null space contains only a zero vector, which, together with Eq. (17), implies that $\frac{\partial [z^{(m)}]_k}{\partial [\hat{\eta}^{(m)}]_j} = 0$. Consequently, given the generating process Eq. (12) and the invertibility of $g^{(m)}$ and $\hat{g}^{(m)}$ (Condition 4.1-A1), the estimated latent variable $\hat{\mathbf{z}}^{(m)}$ and the true latent variable $\mathbf{z}^{(m)}$ are related via an invertible map, as desired.

□

C.2 PROOF FOR THEOREM 4.4

We present the proof for Theorem 4.4. For ease of reference, we duplicate Condition 4.3 and Theorem 4.4.

Condition 4.3 (Component Identifiability Conditions)**.** Over the domain of $(\mathbf{z}, \boldsymbol{\epsilon})$, for any modality m and any $\boldsymbol{T} \notin \mathcal{P}(d(\mathbf{z}))$, we have

$$\sum_{m\neq n\in[M]} \left\| \boldsymbol{T}_m^{-1} \, [\boldsymbol{G}]_{(m),(n)} \, \boldsymbol{T}_n \right\|_0 > \sum_{m\neq n\in[M]} \left\| [\boldsymbol{G}]_{(m),(n)} \right\|_0. \tag{4}$$

Theorem 4.4 (Component-wise Identifiability)**.** *Let $\boldsymbol{\theta} := (\{g_{\mathbf{x}^{(m)}}, g_{\mathbf{z}^{(m)}}, p(\boldsymbol{\epsilon}^{(m)})\}_{m=1}^M)$ and $\hat{\boldsymbol{\theta}} := (\{\hat{g}_{\mathbf{x}^{(m)}}, \hat{g}_{\mathbf{z}^{(m)}}, \hat{p}(\boldsymbol{\epsilon}^{(m)})\}_{m=1}^M)$ be two specifications of the data-generating process in Eq. (1) and Eq. (2). Suppose that they generate identical observational distributions (i.e., $p(\mathbf{x}) = \hat{p}(\mathbf{x})$) and $\boldsymbol{\theta}$ satisfies Condition 4.1 and Condition 4.3. If $\hat{\boldsymbol{\theta}}$ satisfies the following sparse regularization condition:*

$$\sum_{m\neq n\in[M]} \left\| [\hat{\boldsymbol{G}}]_{(m),(n)} \right\|_0 \leq \sum_{m\neq n\in[M]} \left\| [\boldsymbol{G}]_{(m),(n)} \right\|_0, \tag{5}$$

each component $z_i^{(m)}$ and its counterpart $\hat{z}_{\pi(i)}^{(m)}$ are equivalent up to an invertible map $h(\cdot)$, i.e., $\hat{z}_{\pi(i)}^{(m)} = h(z_i^{(m)})$ under a permutation π over $[d(\mathbf{z}^{(m)})]$.

Proof. Given Theorem 4.2, Condition 4.1 implies that the estimated group-wise latent variable $\hat{\mathbf{z}}^{(m)}$ is related to the true variable $\mathbf{z}^{(m)}$ through an invertible transformation $h^{(m)}$, i.e.,

$$\hat{\mathbf{z}}^{(m)} = h^{(m)}(\mathbf{z}^{(m)}). \tag{18}$$

It follows that the Jacobian matrix $\boldsymbol{T}_{\frac{\partial \hat{\mathbf{z}}}{\partial \mathbf{z}}}$ can be arranged into a block-diagonal matrix, in which diagonal block m corresponds to a Jacobian matrix $\boldsymbol{T}_{\frac{\partial \hat{\mathbf{z}}^{(m)}}{\partial \mathbf{z}^{(m)}}}$. Then, the goal is to prove that these diagonal blocks are actually generalized permutation matrices, whose each column only contains one nonzero entry.

We divide the proof into several steps for the sake of exposition. At step 1, we derive an equivalence relation between the estimation model $(\hat{g}_z, \hat{g}_x)$ and the true model (g_z, g_x). At step 2, we apply Theorem 4.2 to the equivalence to characterize the relation between the true and the estimated graph structure. At step 3, we leverage the sparsity condition (Condition 4.3) to reason about the identifiability of each component $z_i^{(m)}$ for $m \in [M]$ and $i \in [d(z^{(m)})]$.

Step 1. The generating process in Eq. (1) and the subspace identification Eq. (18) imply

$$\hat{g}_z(\hat{\mathbf{z}}, \hat{\boldsymbol{\epsilon}}) = h \circ g_z(\mathbf{z}, \boldsymbol{\epsilon}), \tag{19}$$

where h is defined as the Cartesian product of individual $h^{(m)}$ functions.

Taking partial derivatives w.r.t, z_i of both sides of Eq. (19) yields:

$$\begin{bmatrix} \boldsymbol{G}_{\frac{\partial \hat{\mathbf{z}}}{\partial \hat{\mathbf{z}}}} & \boldsymbol{T}_{\frac{\partial \hat{\mathbf{z}}}{\partial \hat{\boldsymbol{\epsilon}}}} \end{bmatrix} \begin{bmatrix} \boldsymbol{T}_{\frac{\partial \hat{\mathbf{z}}}{\partial \mathbf{z}}} \\ \boldsymbol{T}_{\frac{\partial \hat{\boldsymbol{\epsilon}}}{\partial \mathbf{z}}} \end{bmatrix} = \boldsymbol{T}_{\frac{\partial \hat{\mathbf{z}}}{\partial \mathbf{z}}} \boldsymbol{G}_{\frac{\partial \mathbf{z}}{\partial \mathbf{z}}}. \tag{20}$$

Each $\boldsymbol{T}$ matrix is the Jacobian matrix consisting of the corresponding partial derivatives. We use $\boldsymbol{G}_{\frac{\partial \mathbf{z}}{\partial \mathbf{z}}}$ to denote the derivatives from the function g_z which encodes the dependence structure among z components. The same applies to $\boldsymbol{G}_{\frac{\partial \hat{\mathbf{z}}}{\partial \hat{\mathbf{z}}}}$. As discussed above, the matrix $\boldsymbol{T}_{\frac{\partial \hat{\mathbf{z}}}{\partial \mathbf{z}}}$ has a block-diagonal structure (after proper permutations) with block m corresponding to the Jacobian matrix of $h^{(m)}$. Moreover, the matrix $\boldsymbol{T}_{\frac{\partial \hat{\mathbf{z}}}{\partial \hat{\boldsymbol{\epsilon}}}}$ is strictly diagonal due to the generating function Eq. (1).

Step 2. In this step, we simplify Eq. (20) to derive the relation between the estimated graph structures and true graph structures encoded in $\boldsymbol{G}_{\frac{\partial \hat{\mathbf{z}}}{\partial \hat{\mathbf{z}}}}$ and $\boldsymbol{G}_{\frac{\partial \mathbf{z}}{\partial \mathbf{z}}}$ respectively.

First, we note that the $\boldsymbol{T}_{\frac{\partial \hat{\boldsymbol{\epsilon}}}{\partial \mathbf{z}}}$ is also block-diagonal w.r.t. the groups. To see this, we compute the partial derivatives therein as follows: $\frac{\partial \hat{\epsilon}_i^{(m)}}{\partial z_j^{(n)}} = \frac{\partial \hat{\epsilon}_i^{(m)}}{\partial \tilde{\hat{z}}_i^{(m)}} \frac{\partial \tilde{\hat{z}}_i^{(m)}}{\partial z_j^{(n)}}$, where we denote that output of $\hat{g}_z$ with $\tilde{\hat{z}}$ in the derivative. Due to the equivalent relation $\mathbf{z} = \tilde{\mathbf{z}}$ (Eq. (1)), we have $\frac{\partial \tilde{\hat{z}}_i^{(m)}}{\partial z_j^{(n)}} = \frac{\partial \hat{z}_i^{(m)}}{\partial z_j^{(n)}}$ which is zero for distinct groups $m \neq n$ (Eq. (18)). It follows that

$$\frac{\partial \hat{\epsilon}_i^{(m)}}{\partial z_j^{(n)}} = 0, \; m \neq n. \tag{21}$$

Therefore, we have shown that $\boldsymbol{T}_{\frac{\partial \hat{\boldsymbol{\epsilon}}}{\partial \mathbf{z}}}$ is block-diagonal w.r.t. the groups.

This structure allows us to simplify Eq. (20) to directly characterize the relation between the two graphical structures $\boldsymbol{G}_{\frac{\partial \hat{\mathbf{z}}}{\partial \hat{\mathbf{z}}}}$ and $\boldsymbol{G}_{\frac{\partial \mathbf{z}}{\partial \mathbf{z}}}$. In particular, since $\boldsymbol{T}_{\frac{\partial \hat{\boldsymbol{\epsilon}}}{\partial \mathbf{z}}}$ is block-diagonal and $\boldsymbol{T}_{\frac{\partial \hat{\mathbf{z}}}{\partial \hat{\boldsymbol{\epsilon}}}}$ is diagonal, the off-diagonal blocks on the left-hand side of Eq. (20) are determined by $\boldsymbol{G}_{\frac{\partial \hat{\mathbf{z}}}{\partial \hat{\mathbf{z}}}} \boldsymbol{T}_{\frac{\partial \hat{\mathbf{z}}}{\partial \mathbf{z}}}$. Therefore, it follows from Eq. (20):

$$\left[\boldsymbol{G}_{\frac{\partial \hat{\mathbf{z}}}{\partial \hat{\mathbf{z}}}} \boldsymbol{T}_{\frac{\partial \hat{\mathbf{z}}}{\partial \mathbf{z}}}\right]_{(m),(n)} = \left[\boldsymbol{T}_{\frac{\partial \hat{\mathbf{z}}}{\partial \mathbf{z}}} \boldsymbol{G}_{\frac{\partial \mathbf{z}}{\partial \mathbf{z}}}\right]_{(m),(n)}, \; m \neq n, \tag{22}$$

where we adopt subscripts (m) to denote the block for group m.

On account of the block-diagonal structure of $\boldsymbol{T}_{\frac{\partial \hat{\mathbf{z}}}{\partial \mathbf{z}}}$, the left-hand side of Eq. (22) can be expressed as follows:

$$\left[\boldsymbol{G}_{\frac{\partial \hat{\mathbf{z}}}{\partial \hat{\mathbf{z}}}} \boldsymbol{T}_{\frac{\partial \hat{\mathbf{z}}}{\partial \mathbf{z}}}\right]_{(m),(n)} = \left[\boldsymbol{G}_{\frac{\partial \hat{\mathbf{z}}}{\partial \hat{\mathbf{z}}}}\right]_{(m),:} \left[\boldsymbol{T}_{\frac{\partial \hat{\mathbf{z}}}{\partial \mathbf{z}}}\right]_{:,(n)} = \left[\boldsymbol{G}_{\frac{\partial \hat{\mathbf{z}}}{\partial \hat{\mathbf{z}}}}\right]_{(m),(n)} \left[\boldsymbol{T}_{\frac{\partial \hat{\mathbf{z}}}{\partial \mathbf{z}}}\right]_{(n),(n)}. \tag{23}$$

Analogously, the right-hand side of Eq. (22) can be expressed as:

$$\left[\boldsymbol{T}_{\frac{\partial \hat{\mathbf{z}}}{\partial \mathbf{z}}} \boldsymbol{G}_{\frac{\partial \mathbf{z}}{\partial \mathbf{z}}}\right]_{(m),(n)} = \left[\boldsymbol{T}_{\frac{\partial \hat{\mathbf{z}}}{\partial \mathbf{z}}}\right]_{(m),:} \left[\boldsymbol{G}_{\frac{\partial \mathbf{z}}{\partial \mathbf{z}}}\right]_{:,(n)} = \left[\boldsymbol{T}_{\frac{\partial \hat{\mathbf{z}}}{\partial \mathbf{z}}}\right]_{(m),(m)} \left[\boldsymbol{G}_{\frac{\partial \mathbf{z}}{\partial \mathbf{z}}}\right]_{(m),(n)}. \tag{24}$$

It follows from Eq. (22), Eq. (23), and Eq. (24) that

$$\begin{aligned} &\left[\boldsymbol{G}_{\frac{\partial \hat{\mathbf{z}}}{\partial \hat{\mathbf{z}}}}\right]_{(m),(n)} \left[\boldsymbol{T}_{\frac{\partial \hat{\mathbf{z}}}{\partial \mathbf{z}}}\right]_{(n),(n)} = \left[\boldsymbol{T}_{\frac{\partial \hat{\mathbf{z}}}{\partial \mathbf{z}}}\right]_{(m),(m)} \left[\boldsymbol{G}_{\frac{\partial \mathbf{z}}{\partial \mathbf{z}}}\right]_{(m),(n)} \\ \Longrightarrow \; &\left[\boldsymbol{G}_{\frac{\partial \hat{\mathbf{z}}}{\partial \hat{\mathbf{z}}}}\right]_{(m),(n)} = \left[\boldsymbol{T}_{\frac{\partial \hat{\mathbf{z}}}{\partial \mathbf{z}}}\right]_{(m),(m)} \left[\boldsymbol{G}_{\frac{\partial \mathbf{z}}{\partial \mathbf{z}}}\right]_{(m),(n)} \left[\boldsymbol{T}_{\frac{\partial \mathbf{z}}{\partial \hat{\mathbf{z}}}}\right]_{(n),(n)}. \end{aligned} \tag{25}$$

Eq. (25) relates the true off-diagonal ($m \neq n$) structure $\left[\boldsymbol{G}_{\frac{\partial \mathbf{z}}{\partial \mathbf{z}}}\right]_{(m),(n)}$ and its estimated counterpart $\left[\boldsymbol{G}_{\frac{\partial \hat{\mathbf{z}}}{\partial \hat{\mathbf{z}}}}\right]_{(m),(n)}$.

Step 3. We now reason about the component-wise identifiability within each modality through the sparsity of the off-diagonal regions.

The component-wise identifiability is equivalent to that each block sub-matrix $\left[\boldsymbol{T}_{\frac{\partial \hat{\mathbf{z}}}{\partial \mathbf{z}}}\right]_{(m),(m)}$ is a generalized permutation matrix, each row/column of which contains only one nonzero element. Suppose that this was not the case, then it would follow from Eq. (25) and Condition 4.3 that

$$\sum_{m \neq n \in [M]} \left\| \left[\boldsymbol{G}_{\frac{\partial \hat{\mathbf{z}}}{\partial \hat{\mathbf{z}}}}\right]_{(m),(n)} \right\|_0 = \sum_{m \neq n \in [M]} \left\| \left[\boldsymbol{T}_{\frac{\partial \hat{\mathbf{z}}}{\partial \mathbf{z}}}\right]_{(m),(m)} \left[\boldsymbol{G}_{\frac{\partial \mathbf{z}}{\partial \mathbf{z}}}\right]_{(m),(n)} \left[\boldsymbol{T}_{\frac{\partial \mathbf{z}}{\partial \hat{\mathbf{z}}}}\right]_{(n),(n)} \right\|_0 \underbrace{>}_{\text{Condition 4.3}} \sum_{m \neq n \in [M]} \left\| \left[\boldsymbol{G}_{\frac{\partial \mathbf{z}}{\partial \mathbf{z}}}\right]_{(m),(n)} \right\|_0, \tag{26}$$

which would violate the sparsity constraint Eq. (5).

That is, the component $\hat{z}_{\hat{i}}^{(m)}$ cannot functionally influence components in $U^{(m)}$ other than $\mathbf{z}_i^{(m)}$. Therefore, we have shown that each block sub-matrix $\left[\boldsymbol{T}_{\frac{\partial \hat{\mathbf{z}}}{\partial \mathbf{z}}}\right]_{(m),(m)}$ is a generalized permutation matrix. Consequently, we have a bijection $\hat{z}_{\hat{i}}^{(m)} = h_{\hat{i}}^{(m)}(z_i^{(m)})$. Since this holds for any group m and any component i, we have arrived at the desired conclusion.

□

C.3 Extended Theorem 4.2 and its Proof

We restate Theorem C.5 from Yao et al. (2023), which we invoke in our Theorem C.7. We drop the entropy regularization term in Yao et al. (2023), since we assume the invertibility of estimated functions $\hat{g}^{(m)}$ directly.

Definition C.1 (View-Specific Encoders)**.** The *view-specific encoders* $R := \{r_k : \mathcal{X}_k \to \mathcal{Z}_{S_k}\}_{k \in V}$ consist of smooth functions mapping from the respective observation spaces to the view-specific latent space, where the dimension of the kth latent space $|S_k|$ is assumed known for all $k \in V$.

Definition C.2 (Selection)**.** A selection $\oslash$ operates between two vectors $a \in \{0,1\}^d, b \in \mathbb{R}^d$ s.t.

$$a \oslash b := [b_j : a_j = 1, j \in [d]]$$

Definition C.3 (Content Selectors)**.** The content selectors $\Phi := \{\phi(i,k)\}_{V_i \in \mathcal{V}, k \in V_i}$ with $\phi^{(i,k)} \in \{0,1\}^{|d(\mathbf{z}^{(m)})|}$ perform selection C.2 on the encoded information: for any subset $V_i \subset [M]$ and view $k \in V_i$ we have the selected representation: $\phi(i,m) \oslash \hat{\mathbf{z}}^{(m)}$ with $\left\|\phi^{(i,k)}\right\|_0 = \left\|\phi^{(i,k')}\right\|_0$ for all $V_i \in \mathcal{V}, k, k' \in V_i$.

Definition C.4 (Information-Sharing Regularizer)**.** The following regularizer penalizes the ℓ_0-norm $\|\cdot\|_0$ of the content selectors Φ: $\operatorname{Reg}(\Phi) := -\sum_{V_i \in \mathcal{V}} \sum_{k \in V_i} \left\|\phi^{(i,k)}\right\|_0$.

Theorem C.5 (View-Specific Encoder for Identifiability (Yao et al., 2023))**.** *Let $R := \{\hat{g}_{(m)}\}_{m=1}^M$ and Φ respectively be the generating functions and content selectors (Definition C.3) that solve the following constrained optimization problem:*

$$\min \operatorname{Reg}(\Phi) \qquad \textit{subject to:} \qquad R, \Phi \in \arg\min \mathcal{L}_{\textit{alignment}}(R, \Phi), \tag{27}$$

where

$$\mathcal{L}_{\textit{alignment}}(R, \Phi) = \sum_{V_i \in \mathcal{V}} \sum_{\substack{m_1, m_2 \in V_i \\ k < k'}} \mathbb{E}\left[\left\|\phi(i,m_1) \oslash [\hat{g}^{(m_1)}]^{-1}(\mathbf{x}_k) - \phi(i,m_2) \oslash [\hat{g}^{m_2}]^{-1}(\mathbf{x}_{m_2})\right\|_2\right] \tag{28}$$

Then for any subset of modalities $V_i \subset [M]$ and any modality $m \in V_i$, $\phi(i,m) \oslash [\hat{g}^{(m)}]^{-1}$ identifies the shared subspace $\mathbf{z}^{(\cap_{m \in V_i} m)}$.

Definition C.6 (Reconstruction Loss)**.** The following loss penalizes the deviation of the estimate $\hat{\mathbf{x}}$ and its corresponding true counterpart $\mathbf{x}$ in ℓ_2 $L_{\text{recons}} := \mathbb{E}_{\mathbf{x}}(\mathbf{x} - \hat{\mathbf{x}})$.

Additional notations. We slightly abuse the notation to denote both sets and vectors with bold symbols $\mathbf{z}$. Let $\mathbf{z}^{(m\cap n)}$ be the set of latent components shared by modality m and n, i.e., $\mathbf{z}^{(m\cap n)} := \mathbf{z}^{(m)} \cap \mathbf{z}^{(n)}$. Analogously, let $\mathbf{z}^{(m\setminus n)}$ be the set of latent components in modality m that are not shared by n, i.e., $\mathbf{z}^{(m\setminus n)} := \mathbf{z}^{(m)} \setminus \mathbf{z}^{(n)}$.

Theorem C.7 (Generalized Subspace Identifiability). *Let* $\{(g_{\mathbf{x}^{(m)}}, \tilde{g}_{\mathbf{x}^{(-m)}})\}_{m=1}^{M}$ *and* $\{(\hat{g}_{\mathbf{x}^{(m)}}, \hat{\tilde{g}}_{\mathbf{x}^{(-m)}})\}_{m=1}^{M}$ *be two specifications of the generating process Eq. (3) with potentially shared variables* $\mathbf{z}^{(m\cap n)}$ *over any two modalities* m *and* n. *Suppose that they both match the observational distribution* $p(\mathbf{x}) = \hat{p}(\mathbf{x})$ *and satisfy Condition 4.1. Then any subspace* $\hat{\mathbf{z}}^{(m)}$*, shared subspace* $\hat{\mathbf{z}}^{(m\cap n)}$ *and their counterparts* $\mathbf{z}^{(m)}$, $\mathbf{z}^{(m\cap n)}$ *are equivalent up to invertible maps.*

Proof. We note that the latent model with shared latent variables across modalities can still be cast into Equation (3) and satisfies Condition 4.1. As a consequence, Theorem 4.2 gives us the subspace identification for each modality as in the disjoint case. Moreover, we can identify any blocks among modalities thanks to Theorem C.5. This concludes the proof.

□

C.4 Extended Theorem 4.4 and its Proof

Additional notations and discussion. The participation of multiple modalities requires a new definition of the shared blocks in $\mathbf{z}$ since the sharing structure could be nested and various numbers of modalities could share one partition. We partition the entire latent space $\mathbf{z}$ into disjoint blocks $\{\mathbf{z}^{(b)}\}_{b\in B}$, whose components z have exactly the same modality membership $\mathcal{M}(z) := \{m \in [M] : z \in \mathbf{z}^{(m)}\}$. We define the $\mathbf{z}^{H(b)}$ as the smallest (the least components) *identified* block in $\mathbf{z}$ that contains $\mathbf{z}^{(b)}$. In the two-modal case, we have $B = \{(m \cap n), (m \setminus n), (n \setminus m)\}$ and $\mathbf{z}^{H(m\setminus n)} = \mathbf{z}^{(m)}$.

We denote $\mathbf{z}^{(b_1)} \prec \mathbf{z}^{(b_2)}$ if block $\mathbf{z}^{(b_1)}$ is shared by a strict subset of modalities that share $\mathbf{z}^{(b_2)}$, i.e., $\mathcal{M}(\mathbf{z}^{(b_1)}) \subsetneq \mathcal{M}(\mathbf{z}^{(b_2)})$. Therefore, we have either $\mathbf{z}^{H(b)} = \mathbf{z}^{(b)}$ (it is identifiable itself) or $\mathbf{z}^{(b)} \prec \mathbf{z}^{H(b)} \setminus \mathbf{z}^{(b)}$ (it is not identifiable by itself but belongs to an identifiable block $\mathbf{z}^{H(b)}$ together with a more deeply shared block $\mathbf{z}^{H(b)} \setminus \mathbf{z}^{(b)}$). We denote former blocks as $b^+ \in B^+$, i.e., $\mathbf{z}^{H(b^+)} = \mathbf{z}^{(b^+)}$, and the latter blocks as $b^- \in B^- = B \setminus B^+$. In the two-modal case, the modal-specific blocks $\mathbf{z}^{(m\setminus n)}$, $\mathbf{z}^{(n\setminus m)}$ are not identifiable themselves, and we have $\mathbf{z}^{(m\setminus n)} \prec \mathbf{z}^{(m\cap n)}$ and $\mathbf{z}^{(n\setminus m)} \prec \mathbf{z}^{(m\cap n)}$, and $B^+ = \{(m \cap n)\}$ and $B^- = \{(m \setminus n), (n \setminus m)\}$.

We note that all shared blocks $\mathbf{z}^{(b^+)}$ are identified. Thus their bijective indeterminacies are w.r.t, themselves, i.e., $\mathbf{z}^{(b^+)} \mapsto \hat{\mathbf{z}}^{(b^+)}$, which implies the square shape of their indeterminacy matrices $[\boldsymbol{T}_{\frac{\partial \hat{\mathbf{z}}}{\partial \mathbf{z}}}]_{(b^+),(b^+)}$. In contrast, the unidentifiable blocks $\hat{\mathbf{z}}^{(b^-)}$ can potentially receive the influence from all other blocks in its minimal block $\mathbf{z}^{H(b^-)}$. However, $\mathbf{z}^{(b^-)}$ do not influence the complement block $\hat{\mathbf{z}}^{H(b^-)} \setminus \hat{\mathbf{z}}^{(b^-)}$, since $\mathbf{z}^{(b^-)} \prec \mathbf{z}^{H(b^-)} \setminus \mathbf{z}^{(b^-)}$. For instance, $\hat{\mathbf{z}}^{(m\setminus n)}$ may receive influences from $\mathbf{z}^{(m\cap n)}$ and $\mathbf{z}^{(m\setminus n)}$. Consequently, their associated non-trivial indeterminacy matrices are $[\boldsymbol{T}_{\frac{\partial \hat{\mathbf{z}}}{\partial \mathbf{z}}}]_{(b^-),H(b^-)}$ (e.g., $[\boldsymbol{T}_{\frac{\partial \hat{\mathbf{z}}}{\partial \mathbf{z}}}]_{(m\setminus n),(m)}$). Thus, the indeterminacy matrix $\boldsymbol{T}$ can be expressed as $\boldsymbol{T} := \boldsymbol{T}_{\text{on}} + \boldsymbol{T}_{\text{off}}$, where The matrix $\boldsymbol{T}_{\text{on}}$ contains all the on-diagonal square invertible matrices $\boldsymbol{T}_{\text{on}} := \text{diag}(\boldsymbol{T}_{\text{on}}^{1}, \ldots, \boldsymbol{T}_{\text{on}}^{(|B|)})$ and $\boldsymbol{T}_{\text{off}}$ contains all the off-diagonal elements potentially nonzero in the regions $(b, H(b) \setminus b)$ for $b \in B$. We denote this class of matrix $\boldsymbol{T}$ as $\mathcal{T}$. We denote a set of blocks $E(b)$ whose memberships are either a strict superset or do not nest with b's membership $E(b) := \{\tilde{b} \in B | \mathbf{z}^{(b)} \prec \mathbf{z}^{(\tilde{b})} \vee \left(\mathcal{M}(\mathbf{z}^{(b)} \not\subset \mathcal{M}(\mathbf{z}^{(\tilde{b})}) \wedge \mathcal{M}(\mathbf{z}^{(\tilde{b})} \not\subset \mathcal{M}(\mathbf{z}^{(b)})\right)\}$. In the two-modality case, we have $E(m \setminus n) = \{(m \cap n), (n \setminus m)\}$. The regions $\{(E(b), b)\}_{b\in B}$ in the alternative graph $\hat{\boldsymbol{G}}$ reveal identifiability of the latent variables. With these notations, we state the generalized component identification result in Theorem C.9.

Condition C.8 (Generalized Component Identifiability Conditions). Over the domain of $(\mathbf{z}, \boldsymbol{\epsilon})$, for any modality m, for $\boldsymbol{T} \in \mathcal{T}$ and $\boldsymbol{T} \notin \mathcal{P}(d(\mathbf{z}))$, we have

$$\sum_{b\in B, \tilde{b}\in E(b)} \left\| [\boldsymbol{T}^{-1}]_{(\tilde{b}),H(\tilde{b})} [\boldsymbol{G}]_{H(\tilde{b}),(b)} [\boldsymbol{T}]_{(b),(b)} \right\|_0 > \sum_{b\in B, \tilde{b}\in E(b)} \left\| [\boldsymbol{G}]_{(\tilde{b}),(b)} \right\|_0. \tag{29}$$

Notice that at the absence of the shared block $(m \cap n)$, Condition C.8 recovers Condition 4.3 where $E(m) = B \setminus \{m\}$ and $H(m \setminus n) = m$, and $H(n \setminus m) = n$.

Theorem C.9 (Generalized Component-wise Identifiability)**.** *Let* $\boldsymbol{\theta} := (\{g_{\mathbf{x}^{(m)}}, g_{\mathbf{z}^{(m)}}, p(\boldsymbol{\epsilon}^{(m)})\}_{m=1}^{M})$ *and* $\hat{\boldsymbol{\theta}} := (\{\hat{g}_{\mathbf{x}^{(m)}}, \hat{g}_{\mathbf{z}^{(m)}}, \hat{p}(\boldsymbol{\epsilon}^{(m)})\}_{m=1}^{M})$ *be two specifications of the data-generating process in Eq. (1) and Eq. (2) with potentially shared variables* $\mathbf{z}^{(m\cap n)}$ *over any modalities* m *and* n*. Suppose that they generate identical observational distributions (i.e.,* $p(\mathbf{x}) = \hat{p}(\mathbf{x})$*) and* $\boldsymbol{\theta}$ *satisfies Condition 4.1 and Condition C.8. If* $\hat{\boldsymbol{\theta}}$ *satisfies the following condition:*

$$\sum_{b\in B, \tilde{b}\in E(b)} \left\| [\hat{\boldsymbol{G}}]_{(\tilde{b}),(b)} \right\|_0 \leq \sum_{b\in B, \tilde{b}\in E(b)} \left\| [\boldsymbol{G}]_{(\tilde{b}),(b)} \right\|_0, \tag{30}$$

each component $z_i^{(m)}$ *and its counterpart* $\hat{z}_{\pi(i)}^{(m)}$ *are equivalent up to an invertible map* $h(\cdot)$*, i.e.,* $\hat{z}_{\pi(i)}^{(m)} = h(z_i^{(m)})$ *under a permutation* π *over* $[d(\mathbf{z}^{(m)})]$.

Proof. This proof closely follows that of Theorem 4.4. We illustrate the key discrepancies as follows.

We start with only two modalities $\mathbf{z}^{(m)}$ and $\mathbf{z}^{(n)}$ for simplicity and then move on to general cases.

The structure of the indeterminacy matrix $\boldsymbol{T}_{\frac{\partial \hat{\mathbf{z}}}{\partial \mathbf{z}}}$. Identical to Equation 20, we have the relationship between Jacobian matrices:

$$\begin{bmatrix} \boldsymbol{G}_{\frac{\partial \hat{\mathbf{z}}}{\partial \hat{\mathbf{z}}}} & \boldsymbol{T}_{\frac{\partial \hat{\mathbf{z}}}{\partial \hat{\boldsymbol{\epsilon}}}} \end{bmatrix} \begin{bmatrix} \boldsymbol{T}_{\frac{\partial \hat{\mathbf{z}}}{\partial \mathbf{z}}} \\ \boldsymbol{T}_{\frac{\partial \hat{\boldsymbol{\epsilon}}}{\partial \mathbf{z}}} \end{bmatrix} = \boldsymbol{T}_{\frac{\partial \hat{\mathbf{z}}}{\partial \mathbf{z}}} \boldsymbol{G}_{\frac{\partial \mathbf{z}}{\partial \mathbf{z}}}. \tag{31}$$

The presence of the shared block $\mathbf{z}^{(m\cap n)}$ alters the indeterminacy matrix $\boldsymbol{T}_{\frac{\partial \hat{\mathbf{z}}}{\partial \mathbf{z}}}$ – instead of the disjoint diagonal-block shape, $\boldsymbol{T}_{\frac{\partial \hat{\mathbf{z}}}{\partial \mathbf{z}}}$, the columns belonging to the shared variables $\mathbf{z}^{(m\cap n)}$ (shared between two modalities) are possibly nonzero over rows belonging to $\mathbf{z}^{(m\cap n)}$. That is, the shared variables $\mathbf{z}^{(m\cap n)}$ can still mix in the estimates of the two individual parts $\hat{\mathbf{z}}^{(m\setminus n)}$ and $\hat{\mathbf{z}}^{(n\setminus m)}$. However, since we have identified the subspace of $\mathbf{z}^{(m\cap n)}$, its estimates would not contain information of the individual blocks $\mathbf{z}^{(m\setminus n)}$ and $\mathbf{z}^{(n\setminus m)}$, rendering the blocks $\frac{\partial \hat{\mathbf{z}}^{(m\cap n)}}{\partial \mathbf{z}^{(m\setminus n)}} = 0$ and $\frac{\partial \hat{\mathbf{z}}^{(m\cap n)}}{\partial \mathbf{z}^{(n\setminus m)}} = 0$.

The sparse connection among modalities. The reasoning in **Step 2** in the proof of Theorem 4.4 implies that the structure of the matrix $\boldsymbol{T}_{\frac{\partial \hat{\boldsymbol{\epsilon}}}{\partial \mathbf{z}}}$ is consistent with that of the matrix $\boldsymbol{T}_{\frac{\partial \hat{\mathbf{z}}}{\partial \mathbf{z}}}$. That is, they have zero block matrices at the same positions. In particular, since the subspace identifiability in Theorem C.7 implies that the estimated shared variable $\hat{\mathbf{z}}^{(m\cap n)}$ and the modality-specific variable $\hat{\mathbf{z}}^{(n\setminus m)}$ are not influenced by the other modality-specific variables $\mathbf{z}^{(m\setminus n)}$, the same applies to the estimated exogenous variable $\hat{\boldsymbol{\epsilon}}^{(m\cap n)}$ and $\hat{\boldsymbol{\epsilon}}^{(m\setminus n)}$. This structure permits us to disregard $\boldsymbol{T}_{\frac{\partial \hat{\mathbf{z}}}{\partial \hat{\boldsymbol{\epsilon}}}}$ (an identity matrix) and $\boldsymbol{T}_{\frac{\partial \hat{\boldsymbol{\epsilon}}}{\partial \mathbf{z}}}$ on the left-hand side of Eq. (31) when computing a sub-matrix of the right-hand side product:

$$\left[\boldsymbol{G}_{\frac{\partial \hat{\mathbf{z}}}{\partial \hat{\mathbf{z}}}} \boldsymbol{T}_{\frac{\partial \hat{\mathbf{z}}}{\partial \mathbf{z}}}\right]_{(n),(m\setminus n)} = \left[\boldsymbol{T}_{\frac{\partial \hat{\mathbf{z}}}{\partial \mathbf{z}}} \boldsymbol{G}_{\frac{\partial \mathbf{z}}{\partial \mathbf{z}}}\right]_{(n),(m\setminus n)}. \tag{32}$$

We further divide the block $[(n), (m \setminus n)]$ into two blocks along their rows: $[(m \cap n), (m \setminus n)]$ and $[(n \setminus m), (m \setminus n)]$ that represent the influence from $\mathbf{z}^{(m\setminus n)}$ to $\hat{\mathbf{z}}^{(m\cap n)}$ and $\hat{\mathbf{z}}^{(n\setminus m)}$.

Expressing the block $[(m \cap n), (m \setminus n)]$ on the left-hand side of Eq. (32) gives:

$$\left[\boldsymbol{G}_{\frac{\partial \hat{\mathbf{z}}}{\partial \hat{\mathbf{z}}}} \boldsymbol{T}_{\frac{\partial \hat{\mathbf{z}}}{\partial \mathbf{z}}}\right]_{(m\cap n),(m\setminus n)} = \left[\boldsymbol{G}_{\frac{\partial \hat{\mathbf{z}}}{\partial \hat{\mathbf{z}}}}\right]_{(m\cap n),:} \left[\boldsymbol{T}_{\frac{\partial \hat{\mathbf{z}}}{\partial \mathbf{z}}}\right]_{:,(m\setminus n)} = \left[\boldsymbol{G}_{\frac{\partial \hat{\mathbf{z}}}{\partial \hat{\mathbf{z}}}}\right]_{(m\cap n),(m\setminus n)} \left[\boldsymbol{T}_{\frac{\partial \hat{\mathbf{z}}}{\partial \mathbf{z}}}\right]_{(m\setminus n),(m\setminus n)}. \tag{33}$$

Analogously, this block on the right-hand side of Eq. (32) can be expressed as:

$$\left[\boldsymbol{T}_{\frac{\partial \hat{\mathbf{z}}}{\partial \mathbf{z}}} \boldsymbol{G}_{\frac{\partial \mathbf{z}}{\partial \mathbf{z}}}\right]_{(m\cap n),(m\setminus n)} = \left[\boldsymbol{T}_{\frac{\partial \hat{\mathbf{z}}}{\partial \mathbf{z}}}\right]_{(m\cap n),:} \left[\boldsymbol{G}_{\frac{\partial \mathbf{z}}{\partial \mathbf{z}}}\right]_{:,(m\setminus n)} = \left[\boldsymbol{T}_{\frac{\partial \hat{\mathbf{z}}}{\partial \mathbf{z}}}\right]_{(m\cap n),(m\cap n)} \left[\boldsymbol{G}_{\frac{\partial \mathbf{z}}{\partial \mathbf{z}}}\right]_{(m\cap n),(m\setminus n)}. \tag{34}$$

Thus, we have the equality for the block $[(m \cap n), (m \setminus n)]$:

$$\begin{aligned} &\left[\boldsymbol{G}_{\frac{\partial \hat{\mathbf{z}}}{\partial \hat{\mathbf{z}}}}\right]_{(m\cap n),(m\setminus n)} \left[\boldsymbol{T}_{\frac{\partial \hat{\mathbf{z}}}{\partial \mathbf{z}}}\right]_{(m\setminus n),(m\setminus n)} = \left[\boldsymbol{T}_{\frac{\partial \hat{\mathbf{z}}}{\partial \mathbf{z}}}\right]_{(m\cap n),(m\cap n)} \left[\boldsymbol{G}_{\frac{\partial \mathbf{z}}{\partial \mathbf{z}}}\right]_{(m\cap n),(m\setminus n)} \\ &\implies \\ &\left[\boldsymbol{G}_{\frac{\partial \hat{\mathbf{z}}}{\partial \hat{\mathbf{z}}}}\right]_{(m\cap n),(m\setminus n)} = \left[\boldsymbol{T}_{\frac{\partial \hat{\mathbf{z}}}{\partial \mathbf{z}}}\right]_{(m\cap n),(m\cap n)} \left[\boldsymbol{G}_{\frac{\partial \mathbf{z}}{\partial \mathbf{z}}}\right]_{(m\cap n),(m\setminus n)} \left[\boldsymbol{T}_{\frac{\partial \mathbf{z}}{\partial \hat{\mathbf{z}}}}\right]_{(m\setminus n),(m\setminus n)}. \end{aligned} \tag{35}$$

This graphical relation is identical to that in Eq. (25).

However, the relation for the block $[(n \setminus m), (m \setminus n)]$ between two modality-specific parts varies, due to the potential mixing of the shared part into these blocks, which may increase the inbound edges (not outbound edges), as we show below.

For the block $[(n \setminus m), (m \setminus n)]$ on the left-hand side of Eq. (32) gives:

$$\left[\boldsymbol{G}_{\frac{\partial \hat{\mathbf{z}}}{\partial \hat{\mathbf{z}}}} \boldsymbol{T}_{\frac{\partial \hat{\mathbf{z}}}{\partial \mathbf{z}}}\right]_{(n\setminus m),(m\setminus n)} = \left[\boldsymbol{G}_{\frac{\partial \hat{\mathbf{z}}}{\partial \hat{\mathbf{z}}}}\right]_{(n\setminus m),:} \left[\boldsymbol{T}_{\frac{\partial \hat{\mathbf{z}}}{\partial \mathbf{z}}}\right]_{:,(m\setminus n)} = \left[\boldsymbol{G}_{\frac{\partial \hat{\mathbf{z}}}{\partial \hat{\mathbf{z}}}}\right]_{(n\setminus m),(m\setminus n)} \left[\boldsymbol{T}_{\frac{\partial \hat{\mathbf{z}}}{\partial \mathbf{z}}}\right]_{(m\setminus n),(m\setminus n)}. \tag{36}$$

Unlike previous cases, the right-hand side of Eq. (32) for the block involves more than atomic blocks (i.e., it involves the entire modality (n)):

$$\left[\boldsymbol{T}_{\frac{\partial \hat{\mathbf{z}}}{\partial \mathbf{z}}} \boldsymbol{G}_{\frac{\partial \mathbf{z}}{\partial \mathbf{z}}}\right]_{(n\setminus m),(m\setminus n)} = \left[\boldsymbol{T}_{\frac{\partial \hat{\mathbf{z}}}{\partial \mathbf{z}}}\right]_{(n\setminus m),:} \left[\boldsymbol{G}_{\frac{\partial \mathbf{z}}{\partial \mathbf{z}}}\right]_{:,(m\setminus n)} = \left[\boldsymbol{T}_{\frac{\partial \hat{\mathbf{z}}}{\partial \mathbf{z}}}\right]_{(n\setminus m),(n)} \left[\boldsymbol{G}_{\frac{\partial \mathbf{z}}{\partial \mathbf{z}}}\right]_{(n),(m\setminus n)}. \tag{37}$$

Then, it follows from Eq. (36) and Eq. (37) that

$$\begin{aligned} &\left[\boldsymbol{G}_{\frac{\partial \hat{\mathbf{z}}}{\partial \hat{\mathbf{z}}}}\right]_{(n\setminus m),(m\setminus n)} \left[\boldsymbol{T}_{\frac{\partial \hat{\mathbf{z}}}{\partial \mathbf{z}}}\right]_{(m\setminus n),(m\setminus n)} = \left[\boldsymbol{T}_{\frac{\partial \hat{\mathbf{z}}}{\partial \mathbf{z}}}\right]_{(n\setminus m),(n)} \left[\boldsymbol{G}_{\frac{\partial \mathbf{z}}{\partial \mathbf{z}}}\right]_{(n),(m\setminus n)} \\ &\implies \\ &\left[\boldsymbol{G}_{\frac{\partial \hat{\mathbf{z}}}{\partial \hat{\mathbf{z}}}}\right]_{(n\setminus m),(m\setminus n)} = \left[\boldsymbol{T}_{\frac{\partial \hat{\mathbf{z}}}{\partial \mathbf{z}}}\right]_{(n\setminus m),(n)} \left[\boldsymbol{G}_{\frac{\partial \mathbf{z}}{\partial \mathbf{z}}}\right]_{(n),(m\setminus n)} \left[\boldsymbol{T}_{\frac{\partial \mathbf{z}}{\partial \hat{\mathbf{z}}}}\right]_{(m\setminus n),(m\setminus n)}. \end{aligned} \tag{38}$$

We can observe that the existence of the shared variables $\mathbf{z}^{(m\cap n)}$ divides the latent space into finer blocks $\mathbf{z}^{(m\setminus n)}$, $\mathbf{z}^{(n\setminus m)}$, and $\mathbf{z}^{(m\cap n)}$. Eq. (35) and Eq. (38) reveal that the bijective indeterminacy relation hold over these finer blocks, exception for the non-square transition matrix $\left[\boldsymbol{T}_{\frac{\partial \hat{\mathbf{z}}}{\partial \mathbf{z}}}\right]_{(n\setminus m),(n)}$ on the right-hand side of Eq. (38). This is because that the shared part $\mathbf{z}^{(m\cap n)}$ can potentially mix in $\hat{\mathbf{z}}^{(n\setminus m)}$, so $\hat{\mathbf{z}}^{(n\setminus m)}$ may receive edges inbound to $\mathbf{z}^{(m\cap n)}$.

Interplay among multiple modalities. In light of the graphical condition for the two-modality case (Eq. (35) and Eq. (38)), we can derive the conditions for the multi-modality case.

Specifically, we classify the blocks in the estimation graph $\hat{\boldsymbol{G}}_{\frac{\partial \hat{\mathbf{z}}}{\partial \hat{\mathbf{z}}}}$ into the following categories for two distinct atomic blocks b_1 and b_2.

Region 1 : Blocks b_1 and b_2 do not have nested memberships, i.e., $\mathcal{M}(\mathbf{z}^{(b_1)}) \not\subset \mathcal{M}(\mathbf{z}^{(b_2)})$ and $\mathcal{M}(\mathbf{z}^{(b_2)}) \not\subset \mathcal{M}(\mathbf{z}^{(b_1)})$;

Region 2 : Block b_1 has fewer memberships than block b_2: $\mathbf{z}^{(b_1)} \prec \mathbf{z}^{(b_2)}$;

Region 3 : Block b_1 has more memberships than block b_2: $\mathbf{z}^{(b_2)} \prec \mathbf{z}^{(b_1)}$.

Eq. (35) and Eq. (38) reveal that the sparsity for Region 1 and Region 2 is informative, whereas Region 3 is not. This is because in these the inherent indeterminacy from the subspace identifiability within each modality (Theorem 4.2) will engage the product $\boldsymbol{T}_{\frac{\partial \hat{\mathbf{z}}}{\partial \hat{\epsilon}}} \boldsymbol{T}_{\frac{\partial \hat{\epsilon}}{\partial \mathbf{z}}}$ in Eq. (31) in addition to the sparsity in the estimated graph $\boldsymbol{G}_{\frac{\partial \hat{\mathbf{z}}}{\partial \hat{\mathbf{z}}}}$.

Overall conditions. Consolidating all the considerations above, we re-define objects in Condition 4.3 as follows.

1. The indeterminacy matrix $\boldsymbol{T} := \boldsymbol{T}_{\text{on}} + \boldsymbol{T}_{\text{off}}$ is not strictly block-diagonal: The matrix $\boldsymbol{T}_{\text{on}}$ contains all the on-diagonal square invertible matrices $\boldsymbol{T}_{\text{on}} := \text{diag}(\boldsymbol{T}_{b_1}, \ldots, \boldsymbol{T}_{b_{|B|}})$ and $\boldsymbol{T}_{\text{off}}$ contains all the off-diagonal elements potentially nonzero in the regions $(b, H(b) \setminus b)$ for $b \in B$. Also, the matrix multiplication becomes $\left[\boldsymbol{T}_{\frac{\partial \hat{\mathbf{z}}}{\partial \mathbf{z}}}\right]_{(\tilde{b}), H(\tilde{b})} \left[\boldsymbol{G}_{\frac{\partial \mathbf{z}}{\partial \mathbf{z}}}\right]_{H(\tilde{b}),(b)} \left[\boldsymbol{T}_{\frac{\partial \mathbf{z}}{\partial \hat{\mathbf{z}}}}\right]_{(b),(b)}$ as a unified expression of Eq. (35) and Eq. (38).
2. The sub-matrices on which we impose the sparsity controls are exactly the union of Region 2 and Region 1, i.e., the complement of Region 3. We denote such a region as the function of the block index $(E(b), b)$ for each $b \in B$.

With these modifications, the rest of the proof follows exactly from that of Theorem 4.4.

□

D Experimental Details

D.1 Numerical Dataset

We use six numerical datasets in this paper, including three multimodal datasets that satisfy our assumptions and three that slightly violate the sparsity assumptions in the proposed theorems.

Multi-modality settings We generate $n = 10000$ samples according to Eq. (1) and Eq. (2). Following prior work (Von Kügelgen et al., 2021; Yao et al., 2021; Zimmermann et al., 2021), we generate observations using a multi-layer perceptron (MLP). Specifically, the mixing function g is modeled as a three-layer MLP with randomly initialized weights and leaky ReLU activations, enabling g to represent a general nonparametric mixing function. The causal noise terms ϵ are independently and identically distributed (i.i.d.), and the exogenous variables are mutually independent. Sparse inter-modality causal dependencies are randomly generated, ensuring that each modality's latent variables maintain at least one causal connection with another modality.

Ablation settings For the ablation study, we generate two modality observations under different sparsity ratios. Each observation is generated from three causally related latent variables and one exogenous variable. The sample size for each dataset is set to $n = 10000$, and the dimensionality of the observations in each modality is $d(\mathbf{x}) = 20$. The sparsity ratio determines the extent of inter-modality connections among these latent variables. A higher sparsity ratio leads to a sparser causal structure, meaning fewer causal connections between latent variables. Conversely, a lower sparsity ratio yields a denser causal matrix with more causal dependencies. For example, a sparsity ratio of 0% indicates that all inter-modality latent variables are fully connected, whereas a sparsity ratio of 50% implies that half of the possible causal edges are removed.

D.2 Synthetic Dataset

Variant MNIST In real-world scenarios, the ground-truth latent processes are often unknown, making it challenging to evaluate model performance. To address this, we construct a synthetic dataset based on the real image dataset MNIST (LeCun, 1998) with known causal relationships, which supports the setting considered in our work. Our synthetic dataset consists of two modalities, each with latent variables that exhibit causal relationships. The design is flexible. The modalities could correspond to different MNIST variants, such as colored MNIST (Arjovsky et al., 2019) or fashion MNIST (Xiao et al., 2017). The causally related latent variables could be, for example, digit identity, image color, clothing category, image rotation, etc.

In order to make the synthetic setting more intuitive, we introduce an alternative setting: object position acts as a latent variable that influences the appearance of MNIST images. Across different modalities, such causal influence may vary. Furthermore, position in modality 1 may causally influence position in modality 2, which aligns with the data generation process in our work. For

example, the horizontal position of a digit — such as the six — directly influences the transparency of the MNIST image. This horizontal position then serves as a causal factor for the vertical position of shoes in the fashion MNIST, which in turn affects the grayscale intensity of the shoe image. To systematically evaluate the performance of our algorithm under different observational conditions, we consider three variations in colored MNIST, where the digits are assigned one of three colors: red, green, or blue. These relationships are visually illustrated in Figure 7 (a) for clarity.

D.3 Real-world Dataset

In this paper, we consider three types of datasets, including image, time series, and tabular data. Visualizations of the image and time-series datasets are shown in Figure 7 (b-c).

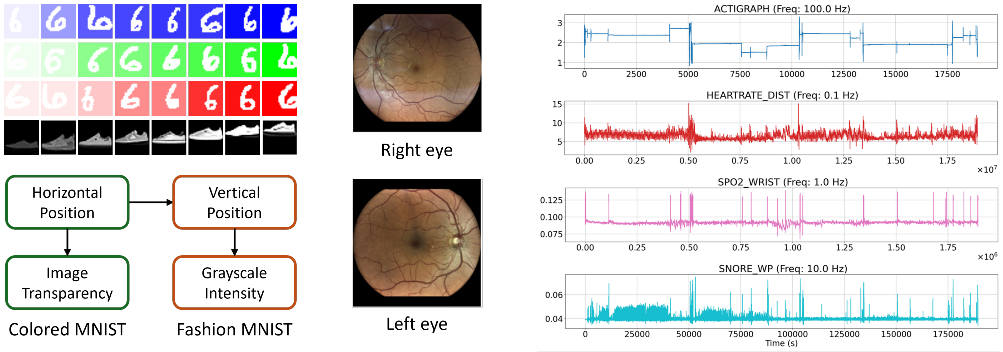

(a) Synthetic dataset (b) Fundus images (c) Recordings on the selected sleep channels

Figure 7: Visualization on the datasets: (a) Synthetic dataset: Variant MNIST. (b) Real-world dataset: Fundus imaging shows the interior surface of the eyes. (c) Real-world dataset: Sleep monitoring shows the time-series recording of sleep-related metrics overnight.

Fundus imaging is the visualization of the interior surface of the fundus, which includes structures such as the optic disc, retina, and retinal microvasculature. High-resolution images of the back of the eye are essential for the diagnosis and monitoring of a variety of eye diseases and conditions.

For example, the retinal microvasculature, which consists of small blood vessels that supply blood to the retina, provides valuable information about eye health. Moreover, fundus imaging can improve understanding of the underlying mechanisms of various eye diseases. It serves as a non-invasive tool to assess the overall health of the microvascular circulation health and provides a direct view of part of the central nervous system.

Sleep monitoring is a time-series dataset collected over three consecutive nights that records various metrics including sleep stage, body position, respiratory events, heart rate, oxygen saturation, and snoring. This dataset focuses on obstructive sleep apnea (OSA), a sleep disorder in which a person's breathing is interrupted during sleep due to the relaxation of throat muscles, causing upper airway obstruction. These interruptions often lead to loud snoring, reduced blood oxygen levels, stress responses, awakenings, and fragmented sleep.

This dataset is collected from a Home Sleep Apnea Test (HSAT), a non-invasive diagnostic method for sleep apnea. Patients wear a portable device overnight to monitor their breathing patterns, heart rate, oxygen levels, snoring, and other sleep patterns. The dataset includes multiple channels, such as ACTIGRAPH for movement, HEARTRATE_DIST for heart rate, SPO2_WRIST for blood oxygen saturation, and SBORE_WP for snoring, capturing key aspects of physical activity and sleep patterns during the HSAT. The device calculates apnea-related indices, including the Apnea/Hypopnea Index (AHI), Respiratory Disturbance Index (RDI), and Oxygen Desaturation Index (ODI), as well as indices for diagnosing conditions such as atrial fibrillation.

D.4 Evaluation Metrics

MCC: Mean Correlation Coefficient MCC is a standard metric used to evaluate the recovery of latent factors in causal representation learning. It measures the alignment between the ground-truth

factors and the estimated latent variables. Specifically, MCC first computes the absolute values of the correlation coefficients between each ground-truth factor and each estimated latent variable. To account for possible permutations of the latent variables, the metric solves a linear sum assignment problem on the computed correlation matrix in polynomial time, ensuring optimal matching between the factors and their corresponding latent representations.

R2: Coefficient of Determination R2 is a standard metric used to evaluate the goodness of fit in regression models. It measures the proportion of variance in the dependent variable that is explained by the independent variables in the model. Specifically, R2 compares the residual sum of squares of the model with the total sum of squares and returns a value between 0 and 1. A higher R2 indicates that the model explains a larger portion of the variance in the data, with 1 representing a perfect fit and 0 indicating that the model explains none of the variability.

SHD: Structural Hamming Distance SHD is a widely used metric for evaluating the accuracy of graph structure recovery in causal discovery. It quantifies the difference between the true causal graph and the estimated graph. Specifically, SHD counts the number of edge modifications—additions, deletions, or reversals—required to transform the estimated graph into the ground-truth graph. This metric provides a simple yet effective measure of structural similarity, with a lower SHD indicating a closer alignment between the estimated and true causal structures.

D.5 Detailed Discussion on Human Phenotype

Without learning such latent variables, we cannot provide a causal explanation between different modalities. The estimated model shows all causal influences involved, suggests the existence of hidden causal variables, and illustrates their relationships with each other and with observable data. Asymptotically, the learned adjacency matrix A corresponds to a graph within the Markov equivalence class given by the PC algorithm.

To interpret the learned hidden variables, we primarily refer to the existing medical literature, which supports their alignment with background knowledge, thereby adding validity to our results. For example, the latent variable FRight3 relates handgrip strength to fundus imaging, consistent with findings showing that handgrip strength correlates with intraocular pressure (IOP) (Pérez-Castilla et al., 2021). In addition, the association between the cataract and changes in IOP (Slabaugh et al., 2013) is consistent with the findings of the model. These connections underline the physiological relevance of the learned hidden variable. Similarly, FRight1 and FLeft1, associated with fundus imaging and age estimation, are consistent with studies demonstrating age-related changes in fundus image color content (Ege et al., 2002). Another latent variable Sleep1 associated with oxygen saturation and sleep metrics aligns with findings that oxygen saturation is a strong predictor of obstructive sleep apnea (OSA) severity (Wali et al., 2020). This indicates that the model's latent variable effectively captures critical factors related to sleep disorders.

E Extended Experiment

To further assess the robustness, scalability, and applicability of our proposed method, we conducted a series of extended experiments under more complex scenarios. These experiments aim to evaluate the performance under diverse latent variable configurations, varying sample sizes, and different structural assumptions, including non-DAG settings and shared latent variables.

Performance in complex scenarios. To evaluate the scalability and generalizability of our method to complex causal structures, we conducted additional experiments on higher-dimensional simulated tasks with diverse configurations of latent variables and modalities. These setups introduce significantly more complex causal relationships between variables. Specifically, we consider three extended scenarios: (1) *Five-mods*, with 30-dimensional observations from five modalities with two latent variables and one exogenous variable per modality. (2) *Six-mods*, with 30-dimensional observations from six modalities with two latent variables and one exogenous variable per modality. (3) *Eight-mods*, involving 30-dimensional observations from eight modalities with two latent variables and one exogenous variable per modality. The results, summarized in Table 4, show that our method consistently delivers robust performance under these challenging conditions.

Impact of the number of latent variables. In real-world applications, the true number of latent variables is typically unknown, and arbitrarily predefining this number may introduce bias and degrade model performance. In this section, we discuss how our method can eliminate the redundant effect of the latent variables, and introduce a cross-validation-based method to determine the appropriate number of latent nodes. By manually setting a range for the number of latent variables and selecting the one with the lowest validation loss, we ensure a principled approach that is both simple and widely applicable (Khemakhem et al., 2020b). Here we conduct synthetic experiments to validate its effectiveness. We followed the data generation process in Section D.1, where the ground-truth number of latent variables is two per modality. The results, as shown in Figure 8(a), demonstrate that our approach accurately recovers the correct number of latent variables.

Impact of sample size. To investigate the impact of sample size on model performance, we conducted an additional experiment evaluating the MCC as the number of data samples increased. Following the data generation process described in Section D.1, where the ground-truth number of latent variables is two for two modalities. We systematically increased the sample size from 10,000 to 40,000 and measured MCC and R2 accordingly. The results, presented in Figure 8(b), show a consistent improvement in MCC as the sample size increases. This finding confirms the hypothesis that greater data availability enhances the model's ability to recover the underlying causal structure.

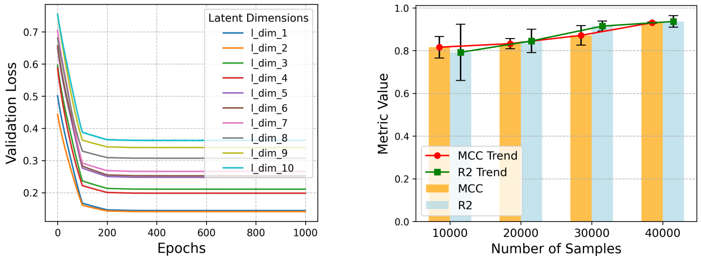

(a) (b)

Figure 8: (a) Comparison of loss across different latent dimensions. (b) The effect of sample size.

Evaluation under non-DAG assumptions. The theoretical results in this paper do not strictly require the assumption of Directed Acyclic Graphs (DAGs) for latent variable structures within or across modalities. To evaluate our method under non-DAG settings, we conducted synthetic experiments where cycles were introduced within and across modalities. Specifically, we followed the data generation process in Section D.1, and considered: (1) cyclic influence within modality; and (2) cyclic influence across modalities. Empirical results in Table 4 demonstrate that the presence of cycles does not hinder the identification of latent variables.

Discussion on the shared latent variables. We present how to preprocess the current framework to accommodate shared variables across modalities and provide empirical results. The extended framework incorporates an additional mechanism to estimate the shared latent variable. Inspired by previous works (Yao et al., 2023; Daunhawer et al., 2023; Von Kügelgen et al., 2021), we incorporate an additional contrastive loss to enforce similarity in the shared latent representations. To evaluate the effectiveness of this extension, we modify the data generation process in Section D.1 and allow for the existence of a shared variable across modalities. The results, summarized in Table 4, show that our method accurately recovers both shared and modality-specific latent variables across different scenarios, confirming the theoretical guarantees of the extended framework.

F Implementation Details

In this section, we provide details of the network architecture, including the optimization scheme and hyperparameter setting.

Metric	Complex Scenarios			Non-DAG Settings		Shared Latent Variables	
	Five mods	Six mods	Eight mods	Cyclic within	Cyclic across	Two mods	Three mods
R2	0.89± 1e-4	0.97± 8e-7	0.83± 1e-3	0.95± 1e-5	0.94± 2e-4	0.86± 5e-4	0.90± 1e-4
MCC	0.84± 3e-4	0.82± 4e-4	0.91± 5e-4	0.89± 2e-4	0.92± 1e-5	0.83±± 7e-4	0.83± 4e-6

Table 4: Extended experiment results across different experimental settings.

F.1 Network Architecture

We summarize our network architecture below and describe it in detail in Table 5.

- **(1,2) Encoder and Decoder**: The encoder transforms raw observations into latent representations, while the decoder reconstructs the inputs from the latent variables. The encoder-decoder design varies depending on the downstream task. For synthetic data, MLPs with leaky ReLU activation were used. For image data, CNN was used as the encoder, and ConvTranspose2D as the decoder. LSTMs were used for time series data. Based on the universal approximation theorem, the model is theoretically able to approximating the underlying mixing function.
- **(3) Learnable Adjacency Matrix**: The causal relationships are embedded in the learned adjacency matrix, where the binary elements indicate whether specific pairs of vertices contribute to the generation of components. It initializes a learnable matrix that captures these dependencies. During the forward pass, the matrix is processed to ensure a directional structure where only certain connections are allowed based on a threshold. This allows the model to learn sparse, meaningful relationships between the latent variables.
- **(4) Flow-based Transformation**: The flow-based transformation is implemented using an MLP to process the latent variable and a flow model for the transformation. The MLP first extracts features from the latent variable, which are then used as input to the flow model, which applies an invertible transformation to the latent space, allowing the model to estimate the noise distribution.

F.2 Training Details

Optimization Scheme. The estimation framework was trained using the Adam optimizer on GPU, and the StepLR scheduler was used to reduce the learning rate periodically. The training process ran for a maximum of 10000 epochs, with early stopping applied if the validation loss does not improve for 20 consecutive epochs. Random seeds were used to ensure reproducibility, and results were averaged across experiments, with variance reported.

The training loss combines multiple components.

- Reconstruction loss: Mean squared error between reconstructed inputs and original data.
- KL divergence loss: Encourages estimated variables to follow a standard normal prior.
- Sparsity loss: An L1-norm penalty is applied to the adjacency matrix to enforce sparsity.

Hyperparameter. The hyperparameters $\alpha = [\alpha_{\text{Ind}}, \alpha_{\text{Sp}}, \alpha_{\text{Recon}}]$ represent the weights assigned to each term in the composite objective function. For each dataset, they were tuned within appropriate logarithmic intervals, ensuring a balance between independence, sparsity, and reconstruction. For the experiments, the following settings were applied: $\alpha = [1e-1, 1e-2, 1]$ for the synthetic dataset, $\alpha = [1e-2, 1e-3, 2]$ for the MNIST dataset, and $\alpha = [1e-1, 1e-2, 1]$ for the phenotype dataset.

G Algorithm Pseudocode

The pseudocode for the proposed algorithm is presented in Algorithm 1.

Configuration	Description	Output
**1.1 MLP-Encoder**	Encoder for numerical data	
Input	Multi-modality observations	BS × d_x
Dense	h_dim neurons, LeakyReLU	BS × h_dim
Dense	h_dim neurons, LeakyReLU	BS × h_dim
Dense	Latent embeddings	BS × l_dim
**2.1 MLP-Decoder**	Decoder for numerical data	
Input	Latent embeddings	BS × l_dim
Dense	h_dim neurons, LeakyReLU	BS × h_dim
Dense	h_dim neurons, LeakyReLU	BS × h_dim
Dense	Reconstructed observations	BS × d_x
**1.2 Image-Encoder**	Encoder for image data	
Input	Image input	BS × 3 × H × W
ResNet18	ResNet backbone, LeakyReLU	BS × h_dim
Dense	Latent embeddings	BS × l_dim
**2.2 Image-Decoder**	Decoder for image data	
Input	Latent embeddings	BS × l_dim
Dense	h_dim neurons	BS × h_dim × H' × W'
ConvTranspose2D	Reconstructed observations	BS × 3 × H × W
**1.3 Time-series Encoder**	Encoder for time-series data	
Input	Multi-channel time-series data	BS × seq_len × n_channel
LSTM	Sequences into hidden representations	BS × h_dim
Output	Latent representation	BS × l_dim
**2.3 Time-series Decoder**	Decoder for time-series data	
Input	Latent representation	BS × l_dim
LSTM	Sequence into output features	BS × seq_len × h_dim
Output	Reconstructed time-series data	BS × seq_len × n_channel
**3. Adjacency Matrix**	Sparsity regularization	
Input	Latent variables from encoders	BS × z_all
Masking	Lower triangular mask	z_all × z_all
Thresholding	Retain entries exceeding threshold	z_all × z_all
Output	Learned causal adjacency matrix	z_all × z_all
**4. Flow Transformation**	Estimate the noise term	
Input	Latent variables across modalities	BS × z_all
Condition Input	Apply adjacency matrix to latent	BS × z_all × z_all
Flow Transformation	Apply transformation to latent	BS × z_all
Output	Estimated noise variables	BS × z_all

Table 5: Architecture details. BS: batch size, d_x: input dimension, l_dim: latent dimension in each modality, z_all: latent dimensions across all modalities, h_dim: hidden dimension, H/W: height/width of the input image, seq_len: sequence length, n_channel: number of channels.

Algorithm 1 Pseudocode for the proposed algorithm.

Input: Grouped observations $\{\mathbf{x}^{(m)}\}_{m=1}^{M}$
Output: Estimated latent variables $\{\hat{\mathbf{z}}^{(m)}\}_{m=1}^{M}$

# Random Initialization
Initialize encoders $\{\text{En}^{(m)}\}_{m=1}^{M}$ and decoders $\{\text{De}^{(m)}\}_{m=1}^{M}$ for each group

# Encoder
Input: Grouped observations $\{\mathbf{x}^{(m)}\}_{m=1}^{M}$
Output: Estimated latent variables $\hat{\mathbf{z}}^{(m)}$ for each group m
for each group $m = 1$ **to** M **do**
    Encode the current group latent and exogenous variables: $\hat{\mathbf{z}}^{(m)}, \hat{\eta}^{(m)} = \text{En}^{(m)}(\mathbf{x}^{(m)})$
end for
Concatenate latent representations: $\{\hat{\mathbf{z}}^{(m)}\}_{m=1}^{M} = \hat{\mathbf{z}}^{(1)} \oplus \hat{\mathbf{z}}^{(2)} \oplus \ldots \oplus \hat{\mathbf{z}}^{(M)}$
return Estimated latent variables and exogenous variables $\{\hat{\mathbf{z}}^{(m)}, \hat{\eta}^{(m)}\}_{m=1}^{M}$

# Flow-based Noise Estimation
Input: Estimated latent variables for each group $\{\hat{\mathbf{z}}^{(m)}\}_{m=1}^{M}$
Output: Estimated noise term $\hat{\epsilon}_{i=1}^{d(\mathbf{z})}$
Initialize adjacency matrix $\hat{\mathbf{A}}$
Select the parents of latent variable based on the adjacency matrix
Pass through flow model to obtain estimated residuals $\hat{\epsilon}_i$
Update the estimated causal graph based on the adjacency matrix with threshold
Compute sparsity loss based on L_1 norm
Compute the KL divergence between $[\{\hat{\eta}^{(m)}\}_{m=1}^{M}, \hat{\epsilon}_{i=1}^{d(\mathbf{z})}]$ and Gaussian prior
return Estimated noise term $\hat{\epsilon}_{i=1}^{d(\mathbf{z})}$

# Decoder
Input: Estimated latent and exogenous variables in each group $\{\hat{\mathbf{z}}^{(m)}, \hat{\eta}^{(m)}\}_{m=1}^{M}$
Output: Reconstructed grouped features $\{\hat{\mathbf{x}}^{(m)}\}_{m=1}^{M}$
for each group $m = 1$ **to** M **do**
    Decode $(\hat{\mathbf{z}}^{(m)}, \hat{\eta}^{(m)})$ to reconstruct features $\hat{\mathbf{x}}^{(m)}$: $\hat{\mathbf{x}}^{(m)} = \text{De}^{(m)}(\hat{\mathbf{z}}^{(m)}, \hat{\eta}^{(m)})$
    Compute reconstruction loss using MSE: $\mathcal{L}_{\text{Recon}}^{(m)} = \text{MSE}(\hat{\mathbf{x}}^{(m)}, \mathbf{x}^{(m)})$
end for
